# Human mesenchymal stem‐derived extracellular vesicles improve body growth and motor function following severe spinal cord injury in rat

**DOI:** 10.1002/ctm2.1284

**Published:** 2023-06-15

**Authors:** Masahito Nakazaki, Karen L. Lankford, Hideaki Yamamoto, Yoshiyuki Mae, Jeffery D. Kocsis

**Affiliations:** ^1^ Department of Neurology Yale University School of Medicine New Haven Connecticut USA; ^2^ VA Connecticut Healthcare System Center for Neuroscience and Regeneration Research West Haven Connecticut USA; ^3^ Department of Neural Regenerative Medicine Research Institute for Frontier Medicine Sapporo Medical University School of Medicine Sapporo Hokkaido Japan; ^4^ Division of Regenerative and Advanced Therapy Nipro Corporation Osaka Osaka Japan

**Keywords:** body growth, extracellular vesicles, mesenchymal stem cells, spinal cord injury

## Abstract

**Background:**

Spinal cord injury (SCI) in young adults leads to severe sensorimotor disabilities as well as slowing of growth. Systemic pro‐inflammatory cytokines are associated with growth failure and muscle wasting. Here we investigated whether intravenous (IV) delivery of small extracellular vesicles (sEVs) derived from human mesenchymal stem/stromal cells (MSC) has therapeutic effects on body growth and motor recovery and can modulate inflammatory cytokines following severe SCI in young adult rats.

**Methods:**

Contusional SCI rats were randomized into three different treatment groups (human and rat MSC‐sEVs and a PBS group) on day 7 post‐SCI. Functional motor recovery and body growth were assessed weekly until day 70 post‐SCI. Trafficking of sEVs after IV infusions in vivo, the uptake of sEVs in vitro, macrophage phenotype at the lesion and cytokine levels at the lesion, liver and systemic circulation were also evaluated.

**Results:**

An IV delivery of both human and rat MSC‐sEVs improved functional motor recovery after SCI and restored normal body growth in young adult SCI rats, indicating a broad therapeutic benefit of MSC‐sEVs and a lack of species specificity for these effects. Human MSC‐sEVs were selectively taken up by M2 macrophages in vivo and in vitro, consistent with our previous observations of rat MSC‐sEV uptake. Furthermore, the infusion of human or rat MSC‐sEVs resulted in an increase in the proportion of M2 macrophages and a decrease in the production of the pro‐inflammatory cytokines tumour necrosis factor‐alpha (TNF‐α) and interleukin (IL)‐6 at the injury site, as well as a reduction in systemic serum levels of TNF‐α and IL‐6 and an increase in growth hormone receptors and IGF‐1 levels in the liver.

**Conclusions:**

Both human and rat MSC‐sEVs promote the recovery of body growth and motor function after SCI in young adult rats possibly via the cytokine modulation of growth‐related hormonal pathways. Thus, MSC‐sEVs affect both metabolic and neurological deficits in SCI.

## Graphical abstract

Spinal cord injury (SCI) in young adults leads to severe sensorimotor disabilities, compounded by the slowing of body growth and muscle loss. Intravenous (IV) infusion of small extracellular vesicles (sEVs) derived from human mesenchymal stem/stromal cells (MSC) restored normal body growth and improved functional motor recovery in young adult SCI rats. An IV delivery of both human and rat MSC‐sEVs resulted in an increase in the proportion of M2 macrophages, a decrease in the pro‐inflammatory cytokine levels of TNF‐α and IL‐6 at the injury site and serum and an increase in the production of growth hormone receptors and IGF‐1 in the liver.

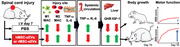

## 1 BACKGROUND

Traumatic spinal cord injury (SCI) results in persistent neurological deficits below the injury level that leads to a host of co‐morbidities.^1^ There are metabolic complications after SCI,^2^ often including a precipitous loss of muscle and bone mass.^3^ Furthermore, the high incidence of SCI in adolescents and young adults who are physiologically transitioning from childhood to adulthood present special challenges related to bone and muscle growth and loss in the young SCI patient population.^4^

Chronic inflammatory diseases in children are associated with growth failure and muscle wasting,^5^ which may result from the suppression of growth hormone receptor (GHR) expression by pro‐inflammatory cytokines.^6^, ^7^ In clinical studies, SCI leads to an increase in pro‐inflammatory cytokines.^8^, ^9^ Here we report that severe SCI in young adult rats results in an increase in pro‐inflammatory cytokines and a reduction in body growth characterized by reduced bone length and muscle mass. Considering the association between systemic pro‐inflammatory cytokines and down‐regulated GHR signalling pathways, we investigated whether a treatment with intravenous (IV) delivery of small extracellular vesicles (sEVs) derived from mesenchymal stem/stromal cells (MSC) known to reduce the production of pro‐inflammatory cytokines after SCI,^10^ mitigates the deleterious effects of SCI on GH signalling and restores a normal growth trajectory in young adult SCI rats.

Intravenous MSC treatment has a therapeutic effect on recovery in experimental models of SCI^11^, ^12^ as does an IV delivery of sEVs (exosomes) derived from cultured MSCs.^10^ Exosomes or sEVs are small (30–150 nm) nanovesicles with bilaminar membranes enriched in sphingolipids and tetraspanin proteins such as CD63 which contain a variety of mRNAs and microRNAs that can be transferred to target cells in an endocrine or paracrine manner.^13^, ^14^ IV‐delivered rat MSC‐sEVs are taken up by M2 macrophages at lesion sites and are associated with an increase in the production of anti‐inflammatory cytokines, such as transforming growth factor‐beta1 (TGF‐β1), stabilization of the microvasculature and improved motor outcome.^10^

Here we show that an IV delivery of both human‐MSC‐derived sEVs (hMSC‐sEVs) and rat MSC–derived sEVs (rMSC‐sEVs) improve functional motor recovery after SCI and restore normal body growth in young adult rats, indicating a broad therapeutic benefit of MSC‐sEVs and a lack of species specificity for these effects. Mechanistic analyses revealed that hMSC‐sEVs are selectively taken up by M2 macrophages in vivo and in vitro, consistent with our previous observations of rat MSC‐sEV uptake.^10^, ^15^ Furthermore, the infusion of hMSC‐sEVs or rMSC‐sEVs results in an increase in the proportion of M2 macrophages and a decrease in the production of the pro‐inflammatory cytokines tumour necrosis factor‐alpha (TNF‐α) and interleukin (IL)‐6 at the injury site, as well as a reduction in systemic serum levels of TNF‐α and IL‐6. GHRs and insulin‐like growth factor 1 (IGF‐1) levels were increased in the liver. These results suggest a mechanism whereby MSC‐sEV treatment decreases systemic pro‐inflammatory cytokines resulting in increased GHRs and IGF‐1 in the liver that may contribute to the normalization of body growth after injury in young adult rats with SCI. SCI commonly results in motor dysfunction and growth‐related complications among young adults. However, IV administration of hMSC‐sEVs treatment exhibits the potential to improve functional motor recovery as well as enhance body growth in this patient population. These results have important therapeutic implications for young SCI patients.

## 2 METHODS

### 2.1 Induction of contusive SCI and assessment of functional recovery

All experiments were carried out in accordance with the National Institutes of Health guidelines for the care and use of laboratory animals, and the VA Connecticut Healthcare System Institutional Animal Care and Use Committee (IACUC) approved all animal protocols.

Contusive SCI was induced in young adult male Sprague–Dawley rats as described previously.^10^, ^12^, ^15^ Briefly, dorsal laminectomies (T9) were performed on adult male Sprague–Dawley rats (190–225 g) under isoflurane gas anaesthesia, followed immediately by a delivery of 22.5 Newton impact (equal to 225 kilodynes) with a 2.5 mm tip using the Infinite Horizon impactor (Precision Systems & Instrumentation, Lexington, KY). All surgical procedures were performed by a single surgeon. Comprehensive details about the surgical procedures conducted on rats for the behaviour analysis are shown in Table S1. Appropriate post‐operative care, including twice‐daily bladder expression for up to 7 days, antibiotic treatment (enrofloxacin 5 mg/kg/day SQ) and pain relief (buprenorphine .05 mg/kg/day SQ) for 48 h, was provided for all animals. Open‐field locomotor function was assessed using the Basso–Beattie–Bresnahan (B–B–B) score^16^ by a tester blinded to the treatment. Rats were scored 2‐day pre‐surgery, 3‐day post‐SCI, 1‐week post‐SCI and at weekly intervals thereafter until the time of sacrifice. In the behaviour analysis, we observed functional recovery through week 10 post‐SCI (study termination period). As in our previous studies using the same SCI model with the same age and severity, we found that SCI rats demonstrated gradual functional recovery until week 7 post‐SCI, followed by a plateau.^10^ Therefore, we concluded that we would be unlikely to observe additional significant motor recovery after week 10 post‐SCI, and that ‘week 10’ was the appropriate time to end the observation period. To assure the consistency of lesions, animals were included in the study only if they had B–B–B scores of 0 on day 3 and .5 on day 7 post‐SCI. A total of 69 surgical animals were used for these experiments with additional nine unoperated controls.

### 2.2 MSC preparation for sEVs

Human bone marrow MSCs were obtained from commercial sources (Lonza, Allendale, NJ), cultured in Dulbecco's Modified Eagle Medium (DMEM) with 10% foetal bovine serum, l‐glutamine and penicillin/streptomycin and passaged six times when cells reached 70%–80% confluency before washing with PBS and transferring to serum‐free media for sEV collection.

Rat MSCs were isolated from the bone marrow of young adult Sprague–Dawley rats (150–200 g) cultured and passaged six times as previously described,^17^ before washing three times with PBS and changing to serum‐free media (DMEM with l‐glutamine and penicillin/streptomycin) for collection of sEVs. Our previous phenotypic analysis of rat MSCs using the same cell preparation methods indicates a CD45^−^, CD106^−^, CD73^+^ and CD90^+^ phenotype consistent with MSCs.^18^

### 2.3 MSC‐sEV isolation and labelling

Exosomes or sEVs fractions were isolated from 2‐day serum‐free conditioned media from human or rat bone marrow cultures using the differential centrifugation method.^10^, ^15^, ^19^ Protein content was assessed using a micro BCA Protein Assay Kit (Thermo Fisher Scientific Inc., Waltham, MA, USA) and Infinite M Plex plate reader (Tecan, San Jose, CA, USA) with comparable protein yields observed for media collected after 48 h from human and rat MSCs.

For experiments tracing EVs trafficking after IV infusion in vivo or uptake of sEVs in vitro, sEVs were labelled with the fluorescence lipophilic tracer, DilC18(7);1,1′‐dioctadecyl‐3,3,3′,3′‐tetramethylindotricbocyanine iodide (DiR) (Molecular Probes, Grand Island, NY, USA) for 10 min according to manufacturer's recommendation, and washed twice with PBS and ultracentrifuged at 100,000 *g* before infusion or addition to cell culture.

### 2.4 Characterization of hMSC‐sEVs

Characterization of rMSC‐sEVs has been described previously.^10^ To characterize the composition of hMSC‐sEVs fractions, a sample of hMSC‐sEVs was sent to Alpha Nano Tech (Morrisville, NC) for transmission electron microscope analysis and particle counts. Copper carbon formvar grids were glow discharged immediately prior to loading with the sample. Sample was processed undiluted. Grid was floated on 10 μL sample drop for 15 min, washed two times with water by floating on the drop of water for 30 s and negatively stained with 2% uranyl acetate by floating on the drop of stain for 30 s. The grid was blot dried with Whatman paper and imaged with Jeol 1230 electron microscope (JEOL, Peabody, MA).

Size distribution of particles in hMSC‐sEVs fractions was also evaluated in the two samples by nanoparticle tracking analysis (NTA) employing SALD‐7500nano (Shimadzu Co., Kyoto, Japan).^10^ Enrichment of hMSC‐sEVs in protein markers of exosomes was assessed in three samples of human hMSC‐sEVs and corresponding hMSCs by western blotting using antibodies directed against exosome makers Alix CD63 and CD9 (see Table S2).

For flow cytometry analysis, samples of isolated hMSC‐sEVs containing 100 μg of total protein in PBS were labelled using a commercial kit (Tetraspanin Exo‐Flow Combo capture kit, System Biosciences) according to the manufacturer's instruction. The hMSC‐sEVs were captured by magnetic beads conjugated with CD9 or CD63 antibodies using a biotin–streptavidin interaction and incubated on ice for 2 h with a FITC‐conjugated antibody. Labelled hMSC‐sEVs were then analysed by a commercial flow cytometer (NovoCyte 3000, ACEA Biosciences) with 488‐nm (blue) laser to assess the surface expression of exosome markers on sEVs.

### 2.5 Infusion of MSC‐sEVs

Previous comparisons of the therapeutic efficacy of rMSC‐sEV treatment efficacy of a fractionated dosing protocol and a single dosing protocol, which both delivered the same total dose of exosomes, showed that the fractionated delivery of sEVs over 3 days was more effective in promoting functional motor recovery after acute SCI rats than a single‐dose protocol.^10^ Based on these findings, we used the 3‐day fractionated treatment protocol in this study to evaluate the relative therapeutic efficacy of hMSC‐sEVs and rMSC‐sEVs. Rats with severe SCI exhibiting a B–B–B score of .5 at day 7 post‐SCI were randomized into three different treatment groups and injected with three equal fractionated doses of hMSC‐sEVs or rMSC‐sEVs suspended in 200 μL PBS, or PBS alone on 3 consecutive days, beginning on day 7 post‐SCI. PBS vehicle or PBS +sEVs was infused via the tail vein in isoflurane‐anaesthetized rats over the course of 1 min with sEV fractions representing a volume of 4.6 μg protein (approximately 2.5 × 10^9^‐hMSC‐sEVs and 2.8 × 10^9^‐rMSC‐sEVs, respectively, as estimated by protein assay and NanoSight LM10, NanoSight Ltd., Minton Park, Amesbury, UK). Experimenters performing infusions were naïve with respect to injection contents, and all subsequent analyses were performed blinded until the data were collected.

To assess the trafficking of hMSC‐sEVs, DiR‐labelled hMSC‐sEVs were administered at five times the standard dose (23 μg total protein hMSC‐sEVs) via the femoral vein of isoflurane‐anaesthetized rats over the course of 1 min, with bupivacaine liposome (5.3 mg/kg SQ) administered post‐surgery for pain relief. Animals infused with DiR‐labelled sEVs were sacrificed after 24 h for histological examination. In our previous studies,^10^, ^15^ we observed the highest concentration of DiR‐labelled MSC‐sEV 24–48 h post‐infusion into our SCI model. Thus, we chose to evaluate the exosome uptake in the SCI model rat at the 48‐h time point.

### 2.6 In vitro uptake of hMSC‐sEVs by macrophages

Macrophages were isolated from the bone marrow of 6‐week‐old SD rats as described in Ying et al.,^20^ plated on 8 well‐multichambered slides (Falcon 354108) at 100,000 cells per well in macrophage growth media consisting of IDMEM without HEPES (Sigma‐Aldrich I3390), with 10% heat‐inactivated foetal calf serum and 10 ng/mL colony‐stimulating factor (CSF) (Sigma‐Aldrich, # SRP3332) and fed every 2–3 days. After 7–10 days, growth media was replaced with M1 or M2 induction media containing either 100 ng/mL lipopolysaccharide (Sigma‐Aldrich, L2630) plus 50 ng/mL interferon‐gamma (Sigma‐Aldrich, I3257) or 10 ng/mL IL‐4 (Sigma‐Aldrich, I3650), respectively, without CSF, at normal pH or a pH of 6. Three days after transfer to M1 or M2 phenotype induction media, 1 μL of a central myelin enriched fraction was added to half of the wells to induce phagocytosis^15^ and DiR‐labelled hMSC‐sEVs were added to wells. After 24 h, cultures were washed three times with plain IDMEM, fixed with 4% paraformaldehyde and stained with antibodies directed against CD206 and iNOS, visualized with fluorescent 488 and 594 wavelength secondary antibodies, counterstained with 4′,6‐diamidino‐2‐phenylindole (DAPI) mounting media, examined, and photographed with a Nikon A1R multiphoton confocal microscope with NIS Elements software as above (Table S2).

### 2.7 Assessment of Post‐SCI growth and feed efficiency

To assess broad physiological recovery post‐SCI in control and sEV‐treated animals, rats receiving the same treatment were pair housed and fed with standard rat chow freely available. Upon group assignment, two rats from each group were housed together in a cage. The body weight of each animal was recorded weekly from the day of surgery until day 70 post‐SCI, along with the weight of any uneaten chow. The body weight measurements were taken for each animal by placing them individually in a designated weighing container. In order to determine the weekly chow consumption, the weight of chow consumed was calculated through the division of the weekly weight of consumed chow by 2, given that consumption was accomplished by paired‐rats. Feed efficiency was calculated by dividing weight gain during the preceding week by the weight of chow consumed during the same period and expressed as a percentage as described.^7^

To assess the effects of treatments on bone growth, body length from the tip of the nose to the base of the tail, length of femoral bones and length and height of cranial bones after cleaning were measured with callipers (VWRi819‐0013, VWR International) in euthanized SCI rats at day 70 post‐SCI. Before the measurement of a femoral bone and a cranial bone, all of the soft tissues around the bones were removed. Body length was measured from the tip of the nose to the base of the tail. Femur length was defined as the distance from the tip of the femoral head to the base of the condyles. The length of cranial bone was measured from the anterior portion of the nasal bones to the most posterior part of the occipital bone and the height of cranial bone was defined as the distance between the sagittal suture and spheno–occipital synchondrosis in the vertical axis.^21^ To assess muscle growth, quadriceps muscle weight was measured at the time of sacrifice (day 70 post‐SCI). Bilateral quadriceps muscles were isolated from femoral bones and weighed.

### 2.8 Histological analysis of sEV localization

Twenty‐four hours after infusion DiR labelled hMSC‐sEVs, rats were deeply anaesthetized with sodium pentobarbital, perfused with saline, followed by 4% paraformaldehyde in .1 M phosphate buffer, and processed for standard frozen sectioning. Coronal (20 μm) sections taken at 1.5 mm from the centre of each block were stained with the primary antibodies directed against macrophage markers, including iNOS and CD206 (Table S2), diluted in .01% Triton X‐100, 5% fish skin gelatin (.1 M PBS) blocking buffer, visualized with species‐specific secondary antibodies (Table S2) counterstained with DAPI mounting media (Vectashield, Vector Laboratories, Burlingame, CA, USA) and photographed with a Nikon A1R multiphoton confocal microscope with NIS Elements software (Nikon, Tokyo, Japan).

### 2.9 Western blot analysis of macrophage markers in the spinal cord and growth hormone receptors in the liver

We assessed local or systemic gene or protein expression at 10‐day post‐SCI (3‐day post‐treatment), as we previously found changes in gene or protein expression induced by intravenous rMSC‐sEVs treatment at this time point.^15^ Three‐day post‐treatment (10‐day post‐SCI), animals were sacrificed under deep anaesthesia with sodium pentobarbital. After extracting a sample of blood for enzyme‐linked immunosorbent assays (ELISA) (see below) and saline perfusion, spinal cords and livers were removed and stored −70°C, and total protein and RNA were purified from a homogenized 8 mm (30 mg wet weight) segment of spinal cord tissue centred around the impact sites or 30 mg liver segment using the AllPrep DNA/RNA/Protein Mini Kit (QIAGEN, Venlo, the Netherlands). Protein concentration was quantified using a BCA protein assay (Thermo Fisher Scientific Inc.), and equal amounts were loaded and run on 10% SDS–polyacrylamide gels (10 μg protein/lane), transferred to polyvinylidene difluoride membranes, probed with appropriate primary antibodies directed against macrophage markers CD206, iNOS, or GHRs (Table S2) overnight at 4°C, followed by secondary antibody staining and visualization with an enhanced chemiluminescence detection system (Pierce Thermo Scientific) using Luminescent Image Analyzer (ChemiDoc MP Imaging System, Bio‐Rad). Band density was analysed using ImageJ software (NIH, Bethesda, MA, USA).

### 2.10 Real‐time quantitative PCR

Total purified RNAs from above‐described samples were quantified with a spectrophotometer (NanoDrop, Technologies Inc., Wilmington, DE, USA), and reverse transcription of RNA was performed with an iScript cDNA Synthesis Kit (Bio‐Rad, Hercules, CA, USA). Quantitative RT‐PCR analysis was performed in triplicate using TaqMan Universal Master Mix II with uracil‐*N* glycosylase (UNG) and specific sets of primers and TaqMan probes (Thermo Fisher Scientific Inc.) (Table S3) with a CFX96TM real‐time PCR detection system (Bio‐Rad), using approximately 20 ng total mRNA for each sample. Thermal cycling was carried out at 50°C for 2 min and 95°C for 10 min, followed by 40 cycles of 95°C for 15 s and 60°C for 1 min. Delta cycle threshold (*Ct*) (Δ*Ct*) was calculated against the endogenous control (glyceraldehyde 3‐phosphate dehydrogenase), and delta–delta *Ct* (ΔΔ*Ct*) was calculated against the Δ*Ct* of the sham. Fold change was calculated using the comparative *Ct* method.^22^

### 2.11 Immunoassay of TNF‐α, IL‐6, GH and IGF‐1

Prior to euthanasia, blood was collected via cardiac puncture after anaesthetizing the rat on day 10 post‐SCI (day 3 post‐treatment) and nonsurgical control rats with same age as the SCI rats. The serum was separated using the centrifugation of the blood at 3500 *g* at 4°C and stored at −80°C until use. TNF‐α was measured using TNF‐α Rat ELISA Kit (BMS622; Thermo Fisher), IL‐6 using Rat IL‐6 ELISA Kit (ERA31RB; Thermo Fisher), GH using Growth Hormone Rat ELISA Kit (KRC5311; Thermo fisher) and IGF‐1 using IGF‐1 Rat ELISA Kit (ERIGF1; Thermo fisher). All data sets used in the analysis are listed in Table S4.

### 2.12 Statistical analysis

All methods and data were reported with the consideration of guidelines provided by the Animals in Research: Reporting in Vivo Experiments^23^ and Minimum Information about a Spinal Cord Injury Experiment.^24^ All statistical analyses were performed using GraphPad Prism software (version 9.0). Repeated‐measures two‐way ANOVA followed by Sidak post hoc tests were conducted for multiple comparisons of B–B–B scores. Continuous data were assessed for normality using the Shapiro–Wilk test. The normally distributed continuous data were analysed by Student's *t*‐test and one‐way analysis of variance, and the Tukey–Kramer test was further used to compare the subgroups. Continuous data that did not pass the normality test were compared using the Mann–Whitney test and the Steel–Dwass test was used to compare the subgroups. All *p* values <.05 were considered statistically significant. All values shown here are expressed as mean ± SEM.

## 3 RESULTS

### 3.1 Characterization of human MSC‐derived sEVs (hMSC‐sEVs)

**FIGURE 1:**
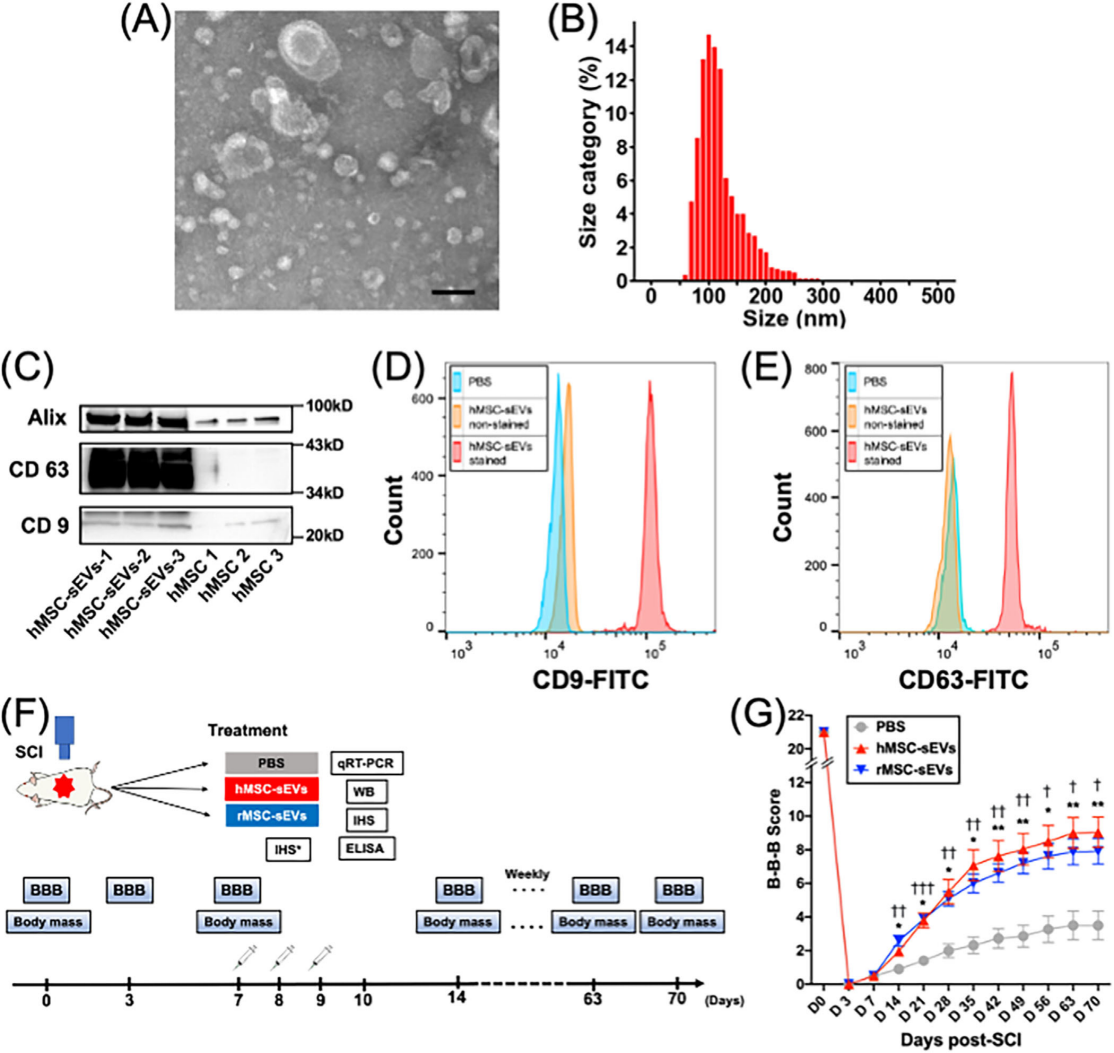
Experimental protocol, characterization of small extracellular vesicles derived from mesenchymal stem/stromal cells (MSC‐sEVs) and effects of MSC‐derived sEVs infusion on functional recovery. (A) Representative electron microscopic image of an sEV preparation showing the sizes and morphology of particles. Scale bar = 100 nm. (B) Histogram of the size distribution of sEVs; (C) expressions of CD 9, CD 63 and Alix in sEVs derived from human MSCs evaluated by western blotting and compared to equal concentrations of proteins from their MSCs; (D and E) expressions of CD 9 and CD 63 in sEVs derived from human MSCs evaluated by flow cytometry. Particle sizes and tetraspanin expression were consistent with sEVs or exosomes; (F) experimental protocol. Animals were randomly assigned to one of three treatment groups: (1) PBS (PBS), (2) human MSC‐derived sEVs (hMSC‐sEVs) and (3) rat MSC–derived sEVs (rMSC‐sEVs), infused with sEVs or PBS over a 3‐day period, beginning at 7‐day post–spinal cord injury (SCI) and evaluated for functional recovery and body weight over a 10 week period or sacrificed for tissue analysis at 10‐day post‐SCI; (G) Basso–Beattie–Bresnahan (B–B–B) locomotor scale scores of contused rats infused with PBS (grey squares, *n* = 11), hMSC‐sEVs (red triangles, *n* = 12) or rMSC‐sEVs (blue upwards triangles, *n* = 12) with three fractionated doses over 3 days, respectively. Values are presented as means ± SEM. Repeated‐measures two‐way ANOVA followed by Sidak post hoc tests were conducted. ^*^ *p* < .05 between hMSC‐sEV and PBS groups, ^**^ *p* < .01 between hMSC‐sEV and PBS groups, ^†^ *p* < .05 between rMSC‐sEV and PBS groups, ^††^ *p* < .01 between rMSC‐sEV and PBS groups. ^†††^ *p* < .001 between rMSC‐sEV and PBS groups. IHS, immunohistochemical staining; qRT‐PCR, quantitative reverse transcription‐polymerase chain reaction; WB, western blotting.

hMSC‐sEVs were isolated from serum‐free culture media of human bone marrow–derived MSCs using a similar differential centrifugation protocol previously employed for the production of rat sEVs^10^ and exhibited the characteristic surface antigen and morphology features of exosomes. Transmission electron microscopy showed that the hMSC‐sEVs samples contained a large number of vesicles in the 70–150 nm diameter size range characteristic of exosomes (Figure 1A) and NTA of samples indicated that the particle sizes ranged from 60 to 200 nm (median 110 mm, *n* = 3 samples) (Figure 1B). These size values were consistent with standard values of MSC‐sEVs^25^ and slightly larger than the particle size distribution we previously reported for rMSC‐sEVs.^10^ Western blots confirmed that hMSC‐sEVs were highly enriched in three characteristic surface marker proteins of exosomes, (CD63, CD9 and Alix), compared to equal amounts of proteins from cultured hMSCs from which the exosomes were obtained (Figure 1C). This level of enrichment of CD63, CD9 and Alix was similar to that previously reported for rMSC‐sEVs^10^ obtained using the same isolation protocol. The presence of exosome‐specific surface maker proteins, CD9 (Figure 1D) and CD63 (Figure 1E), was confirmed by flow cytometry analysis. Collectively, these results are consistent with the hMSC‐sEVs samples containing large numbers of exosomes^26^ and were similar to those we previously described for rMSC‐sEVs.^10^

### 3.2 Infusion of hMSC‐sEVs and rMSC‐sEVs promote comparable levels of locomotor recovery after severe acute SCI

Previously, we reported that the delivery of rMSC‐sEVs in fractionated doses over 3 consecutive days, beginning 1‐week post‐SCI, resulted in significant improvement in functional locomotor recovery in young adult male rats, although delivering the same quantity of rMSC‐sEVs in a single dose did not produce a statistically significant functional improvement.^10^ This suggests that maintaining sEVs levels in the circulation for a more prolonged period of time increases therapeutic efficacy of MSC‐sEVs. We, therefore, used the 3‐day fractionated dosing protocol to assess the therapeutic efficacy of hMSC‐sEVs and compare the efficacy of human versus rat‐derived MSC‐sEVs (Figure 1F). Rats were randomly assigned to one of three treatment groups at 1‐week post‐contusion: PBS, hMSC‐sEV, or rMSC‐sEV groups and injected via tail vein with a total of 4.6 μg of protein from human‐ or rat‐derived MSC‐sEVs delivered in or PBS vehicle only delivered in three equal doses on 3 consecutive days. Open‐field locomotor recovery was evaluated weekly by an investigator who was blinded to the treatment condition, using the B–B–B locomotor rating scale.^16^ In this study, animals in both the hMSC‐sEVs (*n* = 12) and rMSC‐sEVs (*n* = 12) groups showed significantly greater improvement in locomotor recovery compared to the PBS treatment group (*n* = 11). Differences in functional recovery between MSC‐sEV and PBS treatment groups were detectable beginning 1‐week post‐treatment (2‐week post‐SCI), with a relative improvement in recovery increasing over the next several weeks and plateauing at a much higher level in the hMSC‐sEVs and rMSC‐sEVs than the PBS‐treated animals at about week 6 (Figure 1G). Furthermore, the animals in both MSC‐sEVs groups showed a similar pattern of locomotor recovery. At 10‐week post‐SCI, animals in the hMSC‐sEVs and rMSC‐sEVs groups showed average B–B–B scores of 9.04 ± .91 and 7.92 ± .80, respectively, compared to 3.50 ± .86 for the PBS group. Taken together, these findings indicate that hMSC‐sEVs promote functional recovery after SCI with a same degree of efficacy as the same quantity of allogenic rMSC‐sEVs in this rat SCI model.

### 3.3 Young adult rats treated with hMSC‐sEVs or rMSC‐sEVs after SCI showed improved growth relative to vehicle‐treated animals

**FIGURE 2:**
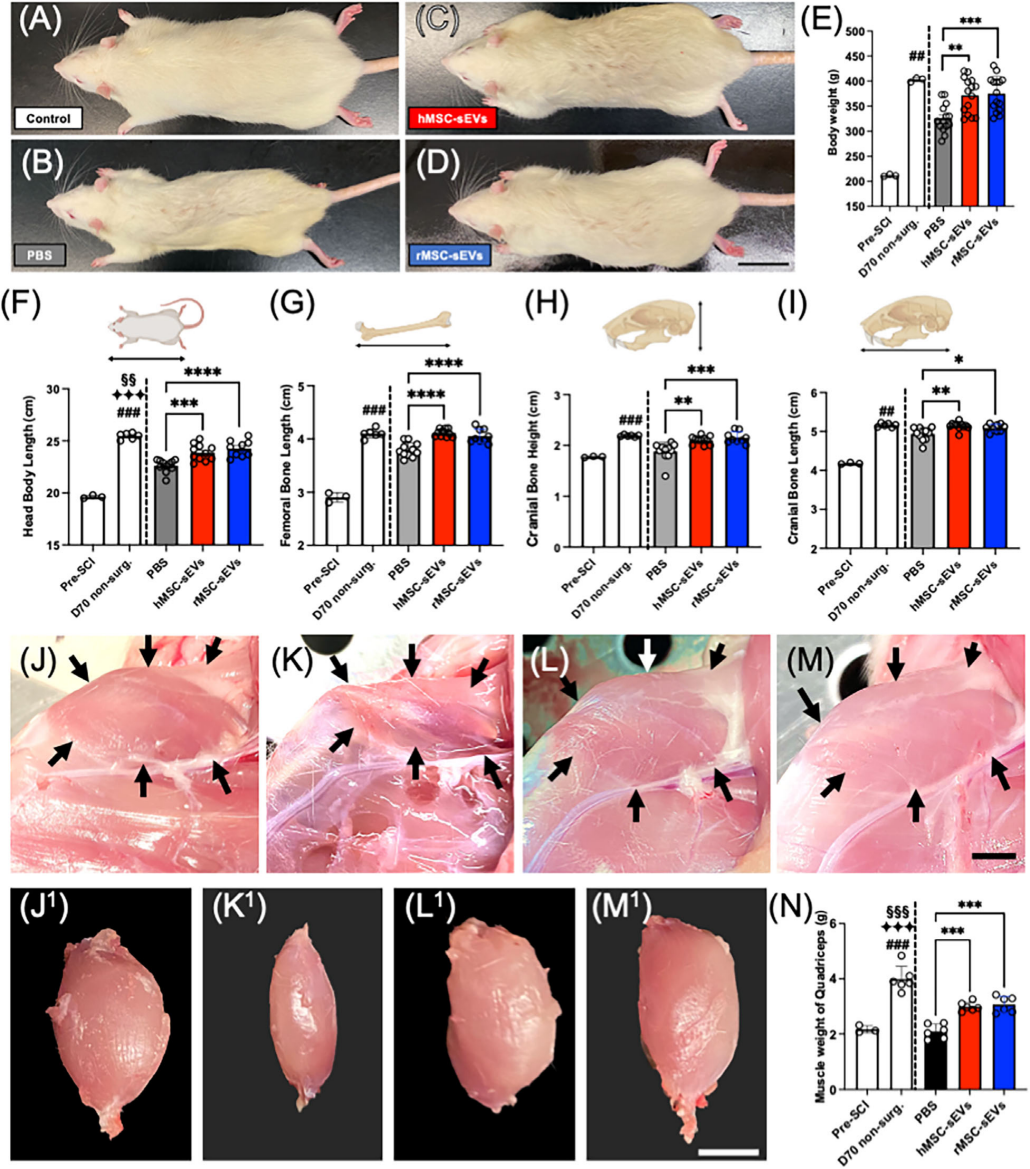
Body growth trajectory after spinal cord injury (SCI). (A–D) Representative images of nonsurgical age‐matched control rats and SCI rats in each group at day 70 post‐SCI – (A) nonsurgical age‐matched control group, (B) PBS group, (C) small extracellular vesicles derived from human mesenchymal stem/stromal cell (hMSC‐sEV) group and (D) sEV derived from rat MSC (rMSC‐sEV) group. Scale bar indicates 3 cm. (E–I) Graphs of body weight (E), head body length (F), femoral bone length (G), cranial bone height (H) and cranial bone length (I), for presurgical animals (*n* = 3), nonsurgical age‐matched controls (*n* = 3) and each treatment group (*n* = 9–12/group) at 70‐day post‐SCI. (J–M) Representative images of in situ quadriceps muscles of age‐matched control rat (J) and SCI rats in the PBS (K) hMSC‐sEVs (L) and rMSC‐sEVs (M) treatment groups at 70‐day post‐SCI. (J^1^–M^1^) Images of isolated quadriceps from parts (J–M). Scale bar indicates 1 cm. (N) Graph of quadriceps muscle weight at day 70 post‐SCI (*n* = 6/group). Values are presented as means ± SEM. A one‐way ANOVA followed by the Tukey–Kramer test or the Kruskal–Wallis test followed by the Steel–Dwass test was conducted. ^*^ *p* < .05, ^**^ *p* < .01, ^***^ *p* < .001, ^****^ *p* < .0001, ^##^ *p* < .01 between nonsurgical age‐matched control group and PBS group, ^###^ *p* < .001 between nonsurgical age‐matched control group and PBS group, ^♦♦♦^ *p* < .001 between nonsurgical age‐matched control group and hMSC‐sEV group, ^§§^ *p* < .01 between nonsurgical age‐matched control group and rMSC‐sEVs group, ^§§§^ *p* < .001 between nonsurgical age‐matched control group and rMSC‐sEVs group.

Animals used in this study, which had not yet reached their full adult size at the start of the experiment, showed evidence of interrupted growth after contusive SCI which was largely reversed by the infusion of MSC‐sEVs. Ten‐week post‐SCI, young adult rats treated with MSC‐sEVs appeared noticeably larger and more robust than SCI animals in the vehicle treatment groups and more similar to nonsurgical age‐matched control (Figure 2A–D). Measurements of body weight at this time point confirmed this visual impression (Figure 2E). The average body weight of PBS‐treated SCI rats was 325.3 ± 8.5 g at 10‐week post‐SCI (a 19% reduction in weight compared to at 406.0 ± 2.4 g for nonsurgical control animals of the same age, *p* = .003). By contrast, mean body weights of hMSC‐sEV‐ and rMSC‐sEV‐treated rats were significantly higher than those of PBS‐treated animals (371.5 ± 10.2 g, 374.9 ± 10.0 g, *p* = .002 and *p* < .001) and did not differ statistically from weights of nonsurgical controls. Although all animals gained weight during the observation period from day 0 (estimated age; 7‐week old) until day 70 post‐SCI (estimated age;17‐week old), SCI rats in the PBS‐treated group gained less weight than the non‐SCI rats (35% of their initial body weight as compared to 47%). The hMSC‐sEVs and rMSC‐sEVs treated rats gained 43% and 44%, respectively, of their initial body weight during the same period (Figure 2E). There was no significant difference in body weight at day 70 post‐SCI between the hMSC‐sEV‐ and rMSC‐sEV‐treated rats.

The more robust appearance and increased body weight in the MSC‐sEV treatment groups relative to the PBS‐treated group corresponded with greater bone growth, both above and below the level of the lesion, in the hMSC‐sEV‐ and rMSC‐sEV‐treated animals compared to the PBS‐treated animals. Total body length, femoral bone length, cranial height and cranial length were all significantly increased in both the hMSC‐sEV and rMSC‐sEV groups compared to the PBS treatment group at 70‐day post‐SCI (Figure 2F–I), although there was no significant difference between the hMSC‐sEV‐ and rMSC‐sEV‐treated rats. Moreover, muscle mass, particularly below the level of the lesion, appeared to be visibly reduced in vehicle‐treated SCI animals at 70‐day post‐SCI compared to either nonsurgical controls or MSC‐sEV‐treated animals. When quadriceps muscles were observed in situ (Figure 2J–M), muscle sizes of SCI animals in the PBS‐treated group (Figure 2K) were noticeably smaller than those in the nonsurgical controls (Figure 2J), whereas those in hMSC‐sEV (Figure 2L) and rMSC‐sEV (Figure 2 M) groups appeared significantly larger than those in the PBS‐treated group (Figure 2K) and similar to sizes in the non‐SCI control group (Figure 2J). These differences were more readily apparent when muscles were dissected (Figure 2N–Q) and confirmed by weighing muscles (Figure 2R). Quadriceps muscle weights of PBS‐treated SCI rats were reduced by an average of 47% compared to nonsurgical control animal (2.10 ± .11 g compared to 3.99 ± .19 g), whereas muscle weights in hMSC‐sEV‐ and rMSC‐sEV‐treated SCI rats were reduced by only 25% and 23%, respectively (2.99 ± .07 g and 3.07 ± .12 g, compared to 3.99 ± .19 g). There was no significant difference between quadriceps weights of hMSC‐sEV‐ and rMSC‐sEV‐treated rats.

### 3.4 Infusion of hMSC‐sEVs or rMSC‐sEVs rapidly restores body growth trajectory

**FIGURE 3:**
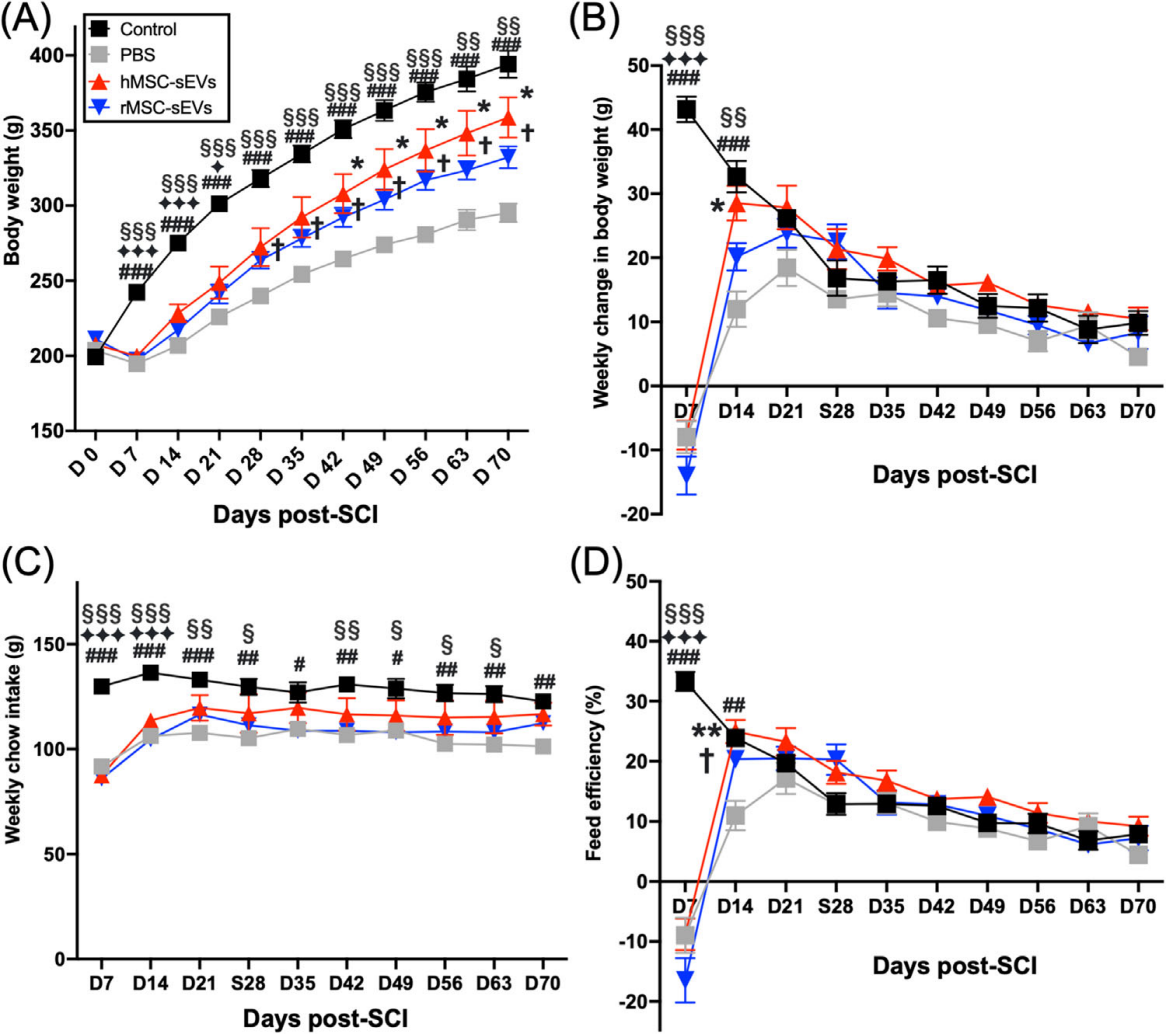
Weekly changes of body weight, chow intake and feed efficiency after spinal cord injury (SCI). (A) Body weight of age‐matched nonsurgical control rats (black squares, *n* = 6) and SCI rats infused with PBS (grey squares, *n* = 8), small extracellular vesicles derived from human mesenchymal stem/stromal cells (hMSC‐sEVs) (red triangles, *n* = 6) or sEVs derived from rat MSC (rMSC‐sEVs) (blue upwards triangles, *n* = 6) with three fractionated doses over 3 days, respectively. (B–D) Weekly change in body weight (B), chow intake (C) and feed efficacy (weight gained/chow consumed) (D) of age‐matched control rats and SCI rats infused with PBS, hMSC‐sEVs or rMSC‐sEVs. Values are presented as means ± SEM. Repeated‐measures two‐way ANOVA followed by Sidak post hoc tests were conducted. ^#^ *p* < .05 between nonsurgical age‐matched control group and PBS group, ^##^ *p* < .01 between nonsurgical age‐matched control group and PBS group, ^###^ *p* < .001 between nonsurgical age‐matched control group and PBS group, ^♦^ *p* < .05 between nonsurgical age‐matched control group and hMSC‐sEVs group,^♦♦♦^ *p* < .001 between nonsurgical age‐matched control group and hMSC‐sEVs group, ^§^ *p* < .05 between nonsurgical age‐matched control group and rMSC‐sEVs group, ^§§^ *p* < .01 between nonsurgical age‐matched control group and rMSC‐sEVs group, ^§§§^ *p* < .001 between nonsurgical age‐matched control group and rMSC‐sEVs group. ^*^ *p* < .05 between hMSC‐sEV and PBS groups, ^**^ *p* < .01 between hMSC‐sEV and PBS groups, ^†^ *p* < .05 between rMSC‐sEV and PBS groups.

Nonsurgical age‐matched control rats in this study showed a gradual increase in body weight throughout the 10‐week observation period (Figure 3A). In the week following SCI, this steady rate of weight gain was disrupted, with a small decrease in body weight being observed 1‐week post‐SCI, and a resumption of weight gain beginning in the second‐week post‐SCI, (Figure 3A). Comparing weekly changes in body weight (Figure 3B), weight gain in nonsurgical control rats gradually decreased during the observation period from 43 ± 1.99 g/week for week 1 and plateauing at about 10 g by weeks 9 and 10. SCI rats, in contrast, showed a negative weight gain during week 1 but returned to a weight gain mode in the following week and eventually matched the weekly weight gains for control rats. Notably, weight gain for SCI animals in the hMSC‐sEVs and rMSC‐sEVs treatment groups returned to values for nonsurgical control animals in the second‐ and third‐week post‐SCI, respectively, whereas PBS‐treated SCI animals matched weight gain for nonsurgical controls beginning only in the fourth week, after weekly weight gains had already declined significantly (Figure 3B).

Weekly weighing of chow intake showed that food consumption for all SCI rats declined sharply during the first‐week post‐SCI but recovered to levels only slightly below nonsurgical controls by the third‐week post‐SCI (Figure 3C). However, there were no differences in food intake between SCI groups at any time point. Feed efficacy (weight gained/chow consumed), an indicator of food metabolism and presumptive GH activity,^7^ did, however, show a difference between treatment groups (Figure 3D). Weight gain resumed more quickly in the MSC‐sEV treatment groups than in the PBS treatment group (Figure 3A,B), but chow intake did not differ between SCI animals (Figure 3C). This indicates that feed efficiency was significantly lower in PBS‐treated SCI rats during the first‐week post‐treatment (second‐week post‐SCI) (Figure 3D) than in either of the MSC‐sEV treatment groups. Thus, the difference in the patterns of weight gain between the different SCI treatment groups could be specifically attributed to the recovery of feed efficiency for the hMSC‐sEV and rMSC‐sEV treatment groups during the first‐week post‐treatment (second‐week post‐SCI) but delayed recovery in the PBS treatment group. There was no significant difference in the patterns of weight gain or feed efficiency between hMSC‐sEV‐ and rMSC‐sEV‐treated rats.

### 3.5 hMSC‐sEVs are taken up by M2 macrophages at the SCI lesion site

**FIGURE 4:**
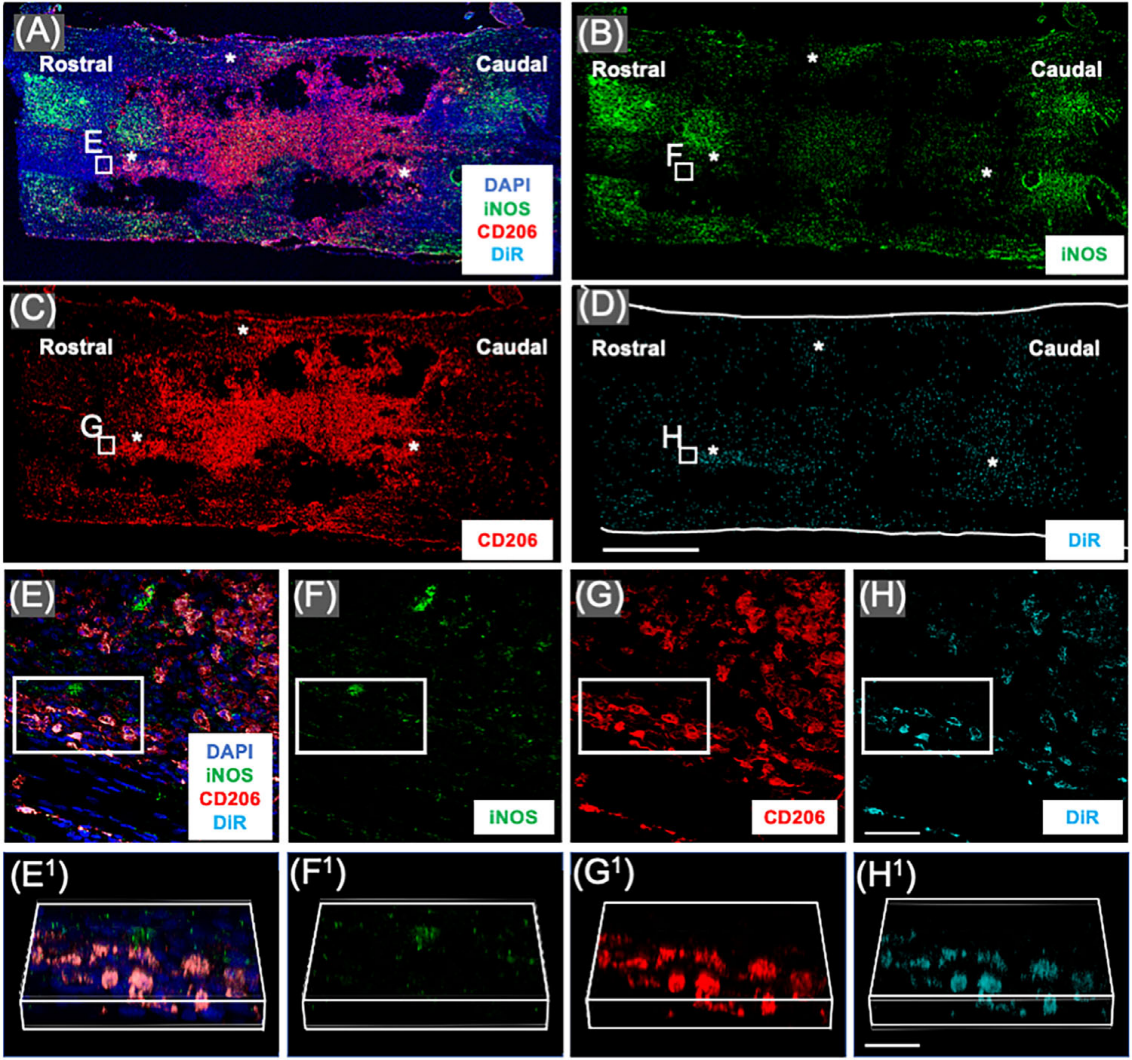
M2 macrophages take up DilC18(7);1,1′‐dioctadecyl‐3,3,3′,3′‐tetramethylindotricbocyanine iodide (DiR)‐labelled human small extracellular vesicles derived from mesenchymal stem/stromal cells (MSC‐sEVs) in vivo at the lesion site after the intravenous infusion. (A–D) Confocal micrographs of a representative region of a frozen sectioned contused spinal cord harvested 48 h after an IV infusion of DiR‐labelled sEVs derived from human MSCs (hMSC‐sEVs), immunostained with antibodies directed against M1 macrophage maker inducible nitric oxide synthase (iNOS) (green), M2 macrophage marker CD206 (red) and 4′,6‐diamidino‐2‐phenylindole (DAPI) (blue) with DiR visualized as cyan. Images show the same area showing fluorescence channels for (A) iNOS, CD206, DAPI and DiR, (B) iNOS, (C) CD206 and (D) DiR. Scale bar in *d* indicates 1 mm. Note that DiR hotspots in DiR‐hMSC‐sEVs infused animals were localized predominantly to cells along the edges of the lesions (‘*’ in parts (A)–(D)). (E–H) Enlarged images of the region boxed area in parts (A)–(D). Images show the same area showing fluorescence channels for (E) iNOS, CD206, DAPI and DiR, (F) iNOS, (G) CD206 and (H) DiR. Images in (E^1^–H^1^) show enlarged images of the boxed area above rotated and illustrated in 3D. Scale bars in (E–H) and (E^1^–H^1^) indicate 20 and 10 μm, respectively.

DiR‐labelled hMSC‐sEVs intravenously delivered 1‐week post‐contusion trafficked to the lesion site and were taken up by a subset of M2 macrophages as previously reported for allogenic DiR‐labelled rMSC‐sEVs.^10^, ^15^ Low magnification images of the lesion sites in SCI rats IV infused with DiR‐labelled hMSC‐sEVs and sacrificed 1 day later showed large numbers of punctate ‘hotspots’ of DiR wavelength fluorescence within the SCI sites and localized predominantly around the periphery of the lesions (Figure 4A–D). High magnification images confirmed that DiR hotspots were localized within CD206+ M2 macrophages (Figure 4E–H), and the intracellular localization of DiR labelling could be verified by rotating z stack images of cells (Figure 4E1–H1). DiR hotspots were not observed in iNOS+ M1 macrophages and were not observed in all CD206 expressing type M2 macrophages, even within the same local area. The observations were consistent in the samples stained with the CD86 antibody for M1 macrophages. No notable presence of DiR hotspots was found in CD86+‐dominant M1 macrophages, whereas a significant prevalence was observed in CD206+ M2 macrophages (Figure S1). These findings indicate that hMSC‐sEVs, MSC‐sEVs from xenogeneic sources, traffic to sites of SCI lesion in the rodent and are taken up by M2 macrophages with a similar pattern as MSC‐sEVs from allogenic sources (rMSC‐sEVs).

### 3.6 Rat bone marrow–derived M2 macrophages take up DiR‐labelled hMSC‐sEVs in vitro

**FIGURE 5:**
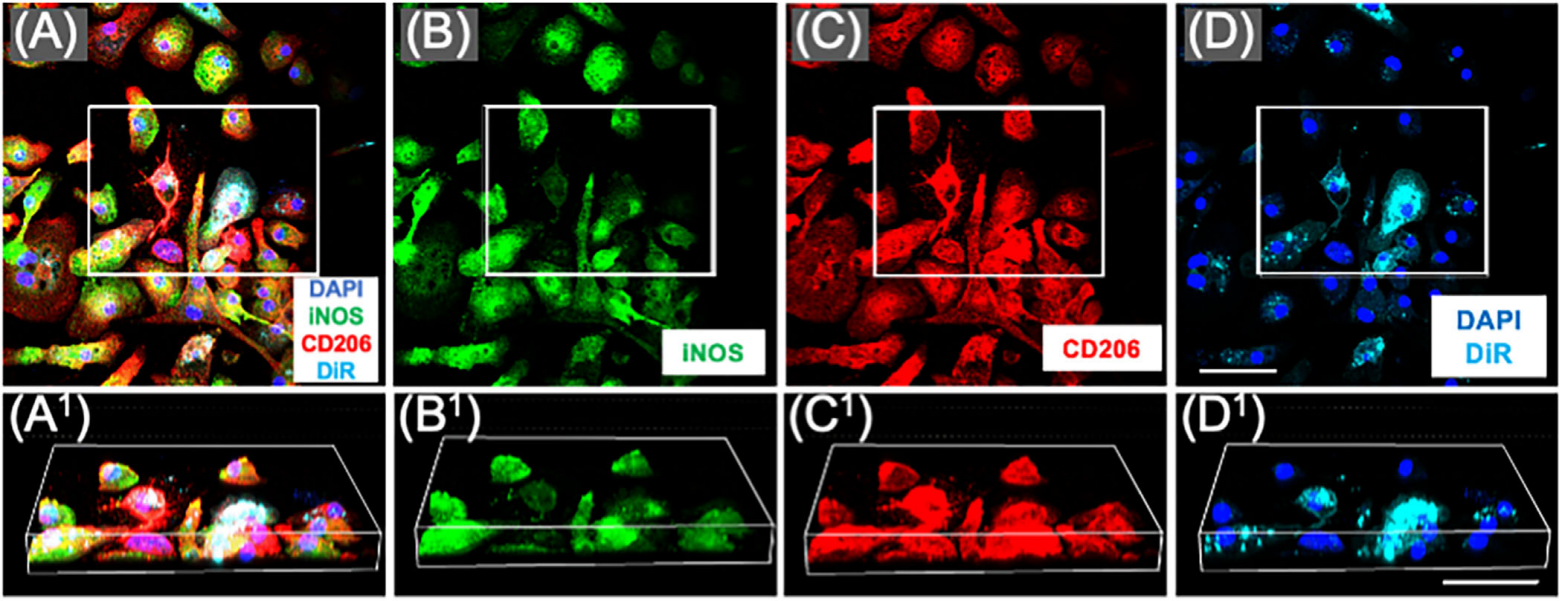
M2 macrophages take up DilC18(7);1,1′‐dioctadecyl‐3,3,3′,3′‐tetramethylindotricbocyanine iodide (DiR)‐labelled human small extracellular vesicles derived from mesenchymal stem/stromal cells (MSC‐sEVs) in vitro. (A–D) Confocal micrographs of a representative region of rat bone marrow macrophage cultures stimulated to induce a phagocytic M2 phenotype with interleukin (IL)‐4/IL‐13 and central myelin enriched fraction and fixed 24 h after the addition of DiR‐labelled sEVs derived from human MSCs (hMSC‐sEVs) at pH 6. Cultures were stained with antibodies directed against CD206 (red), inducible nitric oxide synthase (iNOS) (green) and counterstained with 4′,6‐diamidino‐2‐phenylindole (DAPI) (blue) with DiR fluorescence visualized as cyan. Images from left to right show (A) CD206, iNOS, DAPI and DiR, (B) iNOS, (C) CD206 and (D) DiR and DAPI. Images in (A^1^–D^1^) show enlarged images of the boxed area above rotated and illustrated in 3D. Scale bars in (D) and (D^1^) indicate 50 μm.

As described in greater detail in our previous study,^10^ hotspots of DiR fluorescence were only observed in macrophages induced to an M2 phenotype (Figure 5). Although, in vitro, macrophages expressing the M2 marker CD206 often also expressed the M1 marker iNOS (Figure 5A–C), cells which did not express CD206 were never observed to take up DiR‐hMSC‐sEVs (not shown). This was consistent with our in vivo observations that DiR‐labelled hotspots were only observed within cells expressing CD206 (Figure 4E–H). As in our previous study with rMSC‐sEVs,^10^ low pH conditions and the induction of phagocytic activity by the addition of central myelin enriched fractions greatly increased the uptake of DiR‐hMSC‐sEVs in vitro. However, even under the most favourable conditions, DiR‐hMSC‐sEV uptake was highly variable, and some CD206+ did not show any evidence of DiR‐hMSC‐sEV uptake 24 h after exosome addition (Figure 5) or even after 48 h (not shown).

### 3.7 Both infusion of hMSC‐sEV‐ and rMSC‐sEV‐reduced pro‐inflammatory signals at the SCI lesion site

**FIGURE 6:**
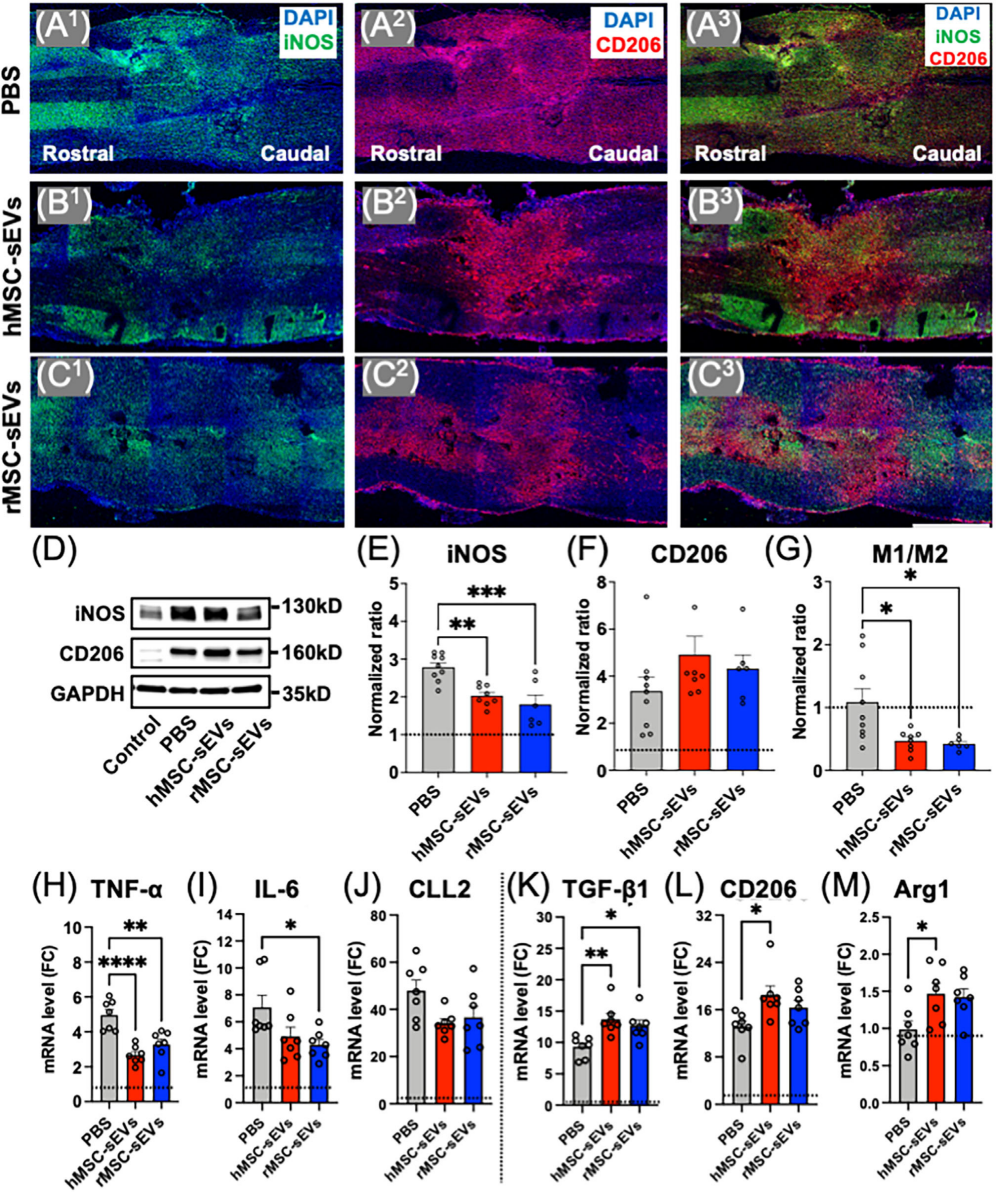
Intravenous infusion of small extracellular vesicles derived from human mesenchymal stem/stromal cells (MSC‐sEVs) and rat MSC‐sEVs promote M2 macrophage polarization in the injured spinal cord. (A^1–3^–C^1–3^) Confocal micrographs of a representative region of a frozen sectioned contused spinal cord harvested 3 days after the treatment (10‐day post–spinal cord injury [SCI]), immunostained with antibodies directed against Type M1 macrophage marker inducible nitric oxide synthase (iNOS) (green), Type M2 macrophage marker CD206 (red) and counterstained with 4′,6‐diamidino‐2‐phenylindole (DAPI) (blue). Scale bar in C^3^ indicates 1 mm; (D) representative western blots of lesion sites harvested at 3‐day post‐treatment (10‐day post‐SCI), immunostained with antibodies directed against iNOS, CD206 and glyceraldehyde 3‐phosphate dehydrogenase (GAPDH); (E–G) graphs of quantitative density analysis for western blot results for iNOS (E) and CD 206 (F) in each treatment condition (*n* = 6–9/group) normalized to control spinal cords protein expression ratios for M1 marker iNOS and M2 marker CD206 (G); (H–J) quantitative reverse transcription‐polymerase chain reaction (qRT‐PCR) analyses of relative mRNA expression levels for pro‐inflammatory cytokines (*n* = 7/group), tumour necrosis factor‐alpha (TNF‐α) (H) and interleukin (IL)‐6 (I), and surface maker for M1 macrophage, CLL2 (J); (K–M) qRT‐PCR analyses of relative mRNA expression levels for an anti‐inflammatory cytokine (*n* = 7/group), TGF‐β1 (K), and makers for M2 macrophage, CD206 (L) and arginase 1 (Arg1) (M). Δ*Ct* was calculated against the endogenous control (GAPDH), and ΔΔ*Ct* (mRNA level) was calculated against the Δ*Ct* of the control. Values are presented as means ± SEM. A one‐way ANOVA followed by the Tukey–Kramer test or the Kruskal–Wallis test followed by the Steel–Dwass test was conducted. ^*^ *p* < .05, ^**^ *p* < .01, ^***^ *p* < .001, ^****^ *p* < .0001. hMSC‐sEVs, small extracellular vesicles derived from human mesenchymal stem/stromal cell; CCL2, C–C motif chemokine ligand 2; Δ*Ct*: delta‐cycle threshold; FC, fold change.

At day 10 post‐SCI (day 3 post‐treatment), immunostaining showed large numbers of macrophages at lesion sites (Figure 6A1–C3). Both macrophages expressing the M1 marker iNOS and the M2 marker CD206 were abundant within the lesion site at this time, with iNOS+ cells being more abundant distal from the epicentre of the lesion (Figure 6A1,B1,C1) and CD206+ cells being located predominantly in the centre of the lesions (Figure 6A2,B2,C2). Compared to the PBS treatment condition (Figure 6A3), animals in both the hMSC‐sEVs (Figure 6B3) and rMSC‐sEVs (Figure 6C3) groups showed a greater proportion of cells staining positive for the M2 marker CD206 relative to the M1 marker iNOS. Quantification of macrophage subtype‐specific proteins confirmed the visual impression of a relative decrease in M1 marker iNOS compared to the M2 marker CD206 in both the hMSC‐sEV and rMSC‐sEV groups on day 10 post‐SCI compared to the PBS‐treated group (Figure 6D–G).

Analysis of gene expression for macrophage‐related cytokines and receptors showed that many macrophage‐related cytokines and receptors investigated were upregulated at the lesion site at day 10 post‐SCI compared to nonsurgical control (Figure 6H–M, dotted line indicates the baseline expression of mRNAs in nonsurgical controls). However, levels of cytokines and receptors differ significantly between treatment groups. Overall, levels of pro‐inflammatory markers (tumour necrosis factor‐alpha (TNF‐α, IL‐6 and CCL2) were lower in the hMSC‐sEV and rMSC‐sEV treatment groups relative to PBS‐treated condition (Figure 6H–J), whereas levels of anti‐inflammatory markers (TGF‐β, CD206 and Arg1) were higher in the hMSC‐sEV and rMSC‐sEV treatment groups (Figure 6K–M). Levels of TNF‐α were significantly lower in both the hMSC‐sEV and rMSC‐sEV groups compared to the PBS group (Figure 6H), whereas IL‐6 levels were significantly lower in the rMSC‐sEV group (Figure 6I), with a trend towards reduction in IL‐6 in the hMSC‐sEV group and lower levels of CCL2 in both MSC‐sEV treatment groups (Figure 6J). In contrast, the level of the anti‐inflammatory cytokines TGF‐β1 was significantly increased in both the hMSC‐sEV and rMSC‐sEV groups at the lesion site compared to the PBS group (Figure 6K) and CD206 (Figure 6L) and arginase 1 (Figure 6 M), and was significantly increased in the hMSC‐sEVs treatment group, with a trend towards increased levels in the rMSC‐sEVs treatment group. Taken together, these data suggest that the infusion of either hMSC‐sEVs or rMSC‐sEVs modulates the inflammatory response at the lesion site after being taken up by M2 macrophages in SCI rats.

### 3.8 Infusion of MSC‐sEVs mitigates effects of SCI on systemic inflammatory cytokines and increases GHR levels in the liver and IGF1 production

**FIGURE 7:**
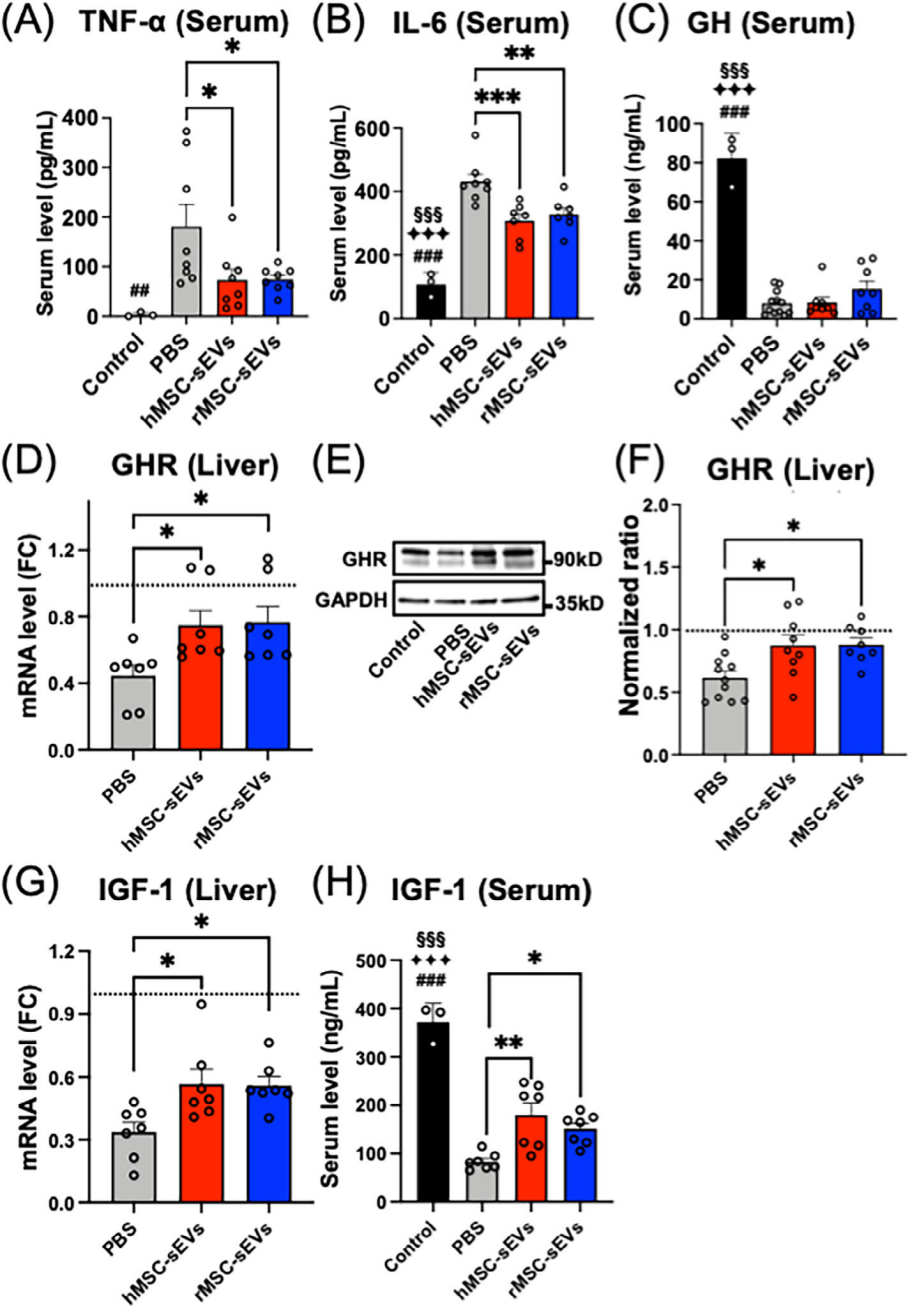
Systemic level of pro‐inflammatory cytokines, growth hormone receptor (GHR) receptors expression on livers and systemic level of insulin‐like growth hormone‐1 (IGF‐1) in spinal cord injury (SCI) rat. (A and B) Graphs of serum level of pro‐inflammatory cytokines, tumour necrosis factor‐alpha (TNF‐α) (A) (*n* = 8/group) and interleukin (IL)‐6 (B) (*n* = 7–8/group); (C) serum levels of growth hormone at day 3 post‐treatment (day 10 post‐SCI) (*n* = 8–12/group); (D–F) levels of GHR mRNA (D) and protein (E and F) in the liver at day 3 post‐treatment (day 10 post‐SCI); quantitative reverse transcription‐polymerase chain reaction (qRT‐PCR) analysis of relative GHR mRNA expression in the liver (D) (*n* = 7/group) and representative western blot (E) and graphical representation (F) of GHR protein levels (F) in liver (*n* = 8–11/group); (G and H) IGF‐1 level in livers and serum at day 3 post‐treatment (day 10 post‐SCI); qRT‐PCR analysis of relative expression of IGF‐1 mRNA in liver (G) (*n* = 7/group) and IGF‐1 protein in serum (H) (*n* = 7/group). Δ*Ct* was calculated against the endogenous control (glyceraldehyde 3‐phosphate dehydrogenase [GAPDH]), and ΔΔ*Ct* (mRNA level) was calculated against the Δ*Ct* of the control. Values are presented as means ± SEM. A one‐way ANOVA followed by the Tukey–Kramer test or the Kruskal–Wallis test followed by the Steel–Dwass test was conducted. ^*^ *p* < .05, ^**^ *p* < .01, ^***^ *p* < .001, ^##^ *p* < .01 between nonsurgical age‐matched control group and PBS group, ^###^ *p* < .001 between nonsurgical age‐matched control group and PBS group, ^♦♦♦^ *p* < .001 between nonsurgical age‐matched control group and small extracellular vesicles derived from human mesenchymal stem/stromal cells (hMSC‐sEVs) group, ^§§§^ *p* < .001 between nonsurgical age‐matched control group and sEV derived from rat mesenchymal stem/stromal cells (rMSC‐sEV) group. ^*^ *p* < .05 between hMSC‐sEVs group and PBS group. FC: fold change.

To investigate a possible mechanism behind the differences in growth pattern between MSC‐sEV and PBS treatment groups after SCI, we evaluated the serum level of the inflammatory cytokines TNF‐α (Figure 7A) and IL6 (Figure 7B) and GH (Figure 7C) after treatment, as well as the effects of treatment on GHR levels in the liver (Figure 7D–F) and potential downstream effects of GH levels on GHRs and (Figure 7D–F) on IGF‐1 (Figure 7G,H) expression. We found that 3‐day post‐treatment (day 10 post‐SCI), SCI rats showed large increases in the serum level of TNF‐α (Figure 7A) and IL‐6 (Figure 7B) compared to nonsurgical control rats, but that serum levels of these pro‐inflammatory cytokines were significantly suppressed in the hMSC‐sEV and rMSC‐sEV groups compared to the PBS group (Figure 7A,B). Serum levels of GH also decreased from 82.19 ± 7.48 ng/mL in nonsurgical controls to 7.99 ± 1.78 ng/mL for PBS, 8.38 ± 2.73 ng/mL for hMSC‐sEV and 15.33 ± 4.09 ng/mL for rMSC‐sEV treatment groups (Figure 7C).

In addition to injury‐induced reductions in serum levels of GH in the SCI rat, both GHR protein levels (Figure 7D,E) and mRNA expression (Figure 7F) were decreased in the liver after SCI. However, the decreases in GHR protein and mRNA in SCI animals were significantly mitigated in SCI rats in the hMSC‐sEV and rMSC‐sEV treatment groups. Likewise, levels of IGF‐1 mRNA in the liver (Figure 7G) and serum levels of IGF‐1 protein (Figure 7H) were dramatically decreased after SCI but significantly restored in the hMSC‐sEV and rMSC‐sEV treatment groups, compared to the PBS treatment group. Thus, MSC‐sEVs treatment mitigated systemic effects of SCI linked to the regulation of growth.

## 4 DISCUSSION

In previous studies, we reported that an IV infusion of MSC‐sEVs derived from rat bone marrow MSCs promoted cellular events, including macrophage‐initiated responses,^15^ resulting in improved functional motor recovery after SCI in rats.^10^ Here we report that severe SCI in young adult rats leads to a profound reduction in body growth, and that IV delivered hMSC‐sEVs promoted body growth normalization as well as improved motor function. These functional changes elicited by MSC‐sEVs were associated with reduction in the pro‐inflammatory cytokines TNF‐α and IL‐6 after SCI at the injury site and in serum. Interestingly, there were also increases in GHR in the liver and IGF‐1 in liver and serum, suggesting a possible mechanism whereby MSC‐sEV treatment improves body growth by modulating GHRs after SCI in young adults, a vulnerable population for SCI.

Dosing and timing of MSC‐sEV delivery were designed to mimic potential levels and timing of sEV release from therapeutically effective infusions of MSCs, which were found to survive in the lungs for about 2–3 days.^12^, ^27^ Moreover, IV‐infused MSC‐sEVs are rapidly cleared through the kidneys,^15^ suggesting that MSC‐sEVs may have a short half‐life in the circulation. We previously reported that rMSC‐sEVs delivered in three fractioned doses over 3 days, beginning 1‐week post‐SCI induction, produced better functional recovery than the delivery of the same quantity in a single dose.^10^ Based on these prior findings using rMSC‐sEVs, we compared the effects of the fractioned delivery of hMSC‐sEVs and rMSC‐sEVs to PBS vehicle at 1‐week post‐SCI. Here we show that IV hMSC‐sEV infusion resulted in significantly better recovery of motor function and body growth than IV PBS infusion and was comparable to improvements produced by rMSC‐sEV infusion, indicating a lack of species specificity for MSC‐sEV therapeutic efficacy. Further studies will be necessary to determine whether increasing MSC‐sEV dosage or treatment period may promote greater recovery.

One of the key advantages of sEV therapy as compared to cell infusion therapy is the more stable and storable properties of sEVs. MSC‐sEVs are also much smaller than the cells from which they are derived, and, unlike cell infusions, sEVs do not lodge in the lungs and risk lung embolisms.^28^ Thus, increased dosages of MSC‐sEVs or repeated dosing with sEVs could be a practical option if such extended treatment is found to be beneficial.

In both human patients and animal models, SCI is associated with broad metabolic consequences in addition to the motor and sensory deficits. Here we show that infusion of MSC‐sEVs rapidly restores the growth trajectory in young adult rodents with SCI. In chronic SCI, many patients exhibit weight gain and metabolic problems related to injury‐induced changes in diet, body composition, metabolic rate and hormonal regulation.^29^ In the acute–subacute phase, body weight decreases after SCI with an increase in body weight being observed significantly later, at about week 26 post‐SCI.^29^ Such early body mass loss might lead to an impaired normal growth trajectory in young adults with SCI.^4^ In the current study, we observed that control‐treated young adult SCI rats showed about 20% reduction in body weight and 12 % reduction in body length compared to nonsurgical age‐matched controls at day 70 post‐SCI. Effects of SCI on growth affected structures above and below the lesion, with statistically significant reductions in femur length, cranial height and width and quadriceps muscle mass. Treatment with MSC‐sEVs, however, resulted in significant improvement in growth, with hMSC‐sEV and rMSC‐sEV groups showing 8% and 7% reductions in body mass, respectively, as compared to 20% in untreated rats. Femur length, cranial height and width and quadriceps muscle mass were also greater in the MSC‐sEV treatment group.

Weekly measurements of body weight and food intake after SCI indicated that the growth differences between MSC‐sEV‐treated and PBS‐treated animals were not due to differences in appetite, but rather to differences in the efficiency of converting food intake into body mass (feed efficiency). All animals showed a reduction in food intake during the first‐week post‐SCI, but there was no difference in weekly food intake among SCI rat groups. However, although feed efficiency was decreased in all SCI treatment groups during the first‐week post‐SCI, both MSC‐sEV groups showed significantly better recovery of feed efficiency during the second‐week post‐SCI (first‐week post‐treatment) as compared to the PBS group. This suggests that changes in metabolic processes affecting feed efficiency, rather than changes in feeding behaviour or appetite,^7^, ^30^ were responsible for the growth deficit in the SCI rats and implicated differences in GH or IGF‐1 signalling as potential causes of growth differences.

Consistent with our previous studies using rat MSC‐derived sEVs,^10^, ^15^ both human‐ and rat‐derived MSC‐sEVs were taken up by a subset of M2 type macrophages. Infusion of MSC‐sEVs from human and rat resulted in a decrease in pro‐inflammatory markers and an increase in anti‐inflammatory markers at lesion site. Notably, the expression of TNF‐α mRNA and IL‐6 were decreased at spinal cord lesion sites in MSC‐sEV‐treated animals compared to PBS‐treated SCI animals, whereas the expression of TGF‐β1 was increased. These changes in the expression of pro‐inflammatory and anti‐inflammatory cytokines at the lesion site correlated with changes in serum levels of the pro‐inflammatory cytokines TNF‐α and IL‐6, both of which were increased 10 days after SCI but significantly decreased in hMSC‐sEV‐ and rMSC‐sEV‐treated SCI animals. These observations suggest that the systemic effects of MSC‐sEVs on growth trajectory might be mediated by MSC‐sEV‐induced changes in local cytokine production leading to changes in systemic levels of cytokines. More specifically, MSC‐sEV uptake by macrophages leads to a reduction in injury‐induced increases in systemic pro‐inflammatory cytokines.

Previous research reported that GHR in the liver is down‐regulated in a cytokine‐dependent manner in rodent models of systemic inflammation.^6^, ^31^, ^32^ In vitro, the signature pro‐inflammatory cytokine, TNF‐α, has been shown to down‐regulate GHR expression by inhibiting Sp1/Sp3 transactivators and block GH‐induced tyrosine phosphorylation of the signal transducer and activator of transcription 5a and 5b (STAT5a/b), which are transcription factors of anabolic target genes, including IGF‐1in rat hepatocytes.^6^, ^33^ Moreover, IL‐6 stimulates SOCS3 and suppresses GH‐induced STAT5b phosphorylation in the liver, causing a failure of GH signalling.^34^ MUP/hIL‐6 transgenic mice overexpressing IL‐6 were also reported to exhibit growth retardation in parallel with a reduction of hepatic GHR mRNA and reduction of serum levels of IGF‐1.^32^ Taken together, the systemic upregulation of the pro‐inflammatory cytokines TNF‐α and IL‐6 in young SCI rats likely contributes to growth reduction by the down‐regulation of GHRs in the liver and reducing serum levels of IGF‐1. The substantial reversal of these SCI‐induced changes in cytokine and hormone levels following MSC‐sEV treatment may explain the more rapid restoration of growth trajectory in MSC‐sEV‐treated animals.

In clinical settings, SCI patients with severe SCI and quadriplegia often exhibit hyposomatomedinemia with low serum levels of GH and IGF‐1, which contributes to a decrease in lean body mass and muscle atrophy and adds further functional impairment to the initial neurological deficit.^35^, ^36^, ^37^ Consistent with these observations in SCI patients, young adult SCI rats in this study showed a significant reduction in both GH (90% reduction) and IGF‐1 (80% reduction) in serum of PBS‐treated SCI rats compared to nonsurgical age‐matched control at day 10 post‐SCI. In addition, serum IGF‐1 (but not GH) was increased in both hMSC‐sEV and rMSC‐sEV treatment groups relative to PBS treatment. This suggests that systemic levels of IGF‐1, but not GH, may directly affect body growth after SCI. This idea is supported by the fact that although GH can act directly on target tissue, many GH effects on the body are mediated indirectly by IGF‐1 that is produced in the liver in response to GH signalling or by interactions between GH and IGF‐1 signalling within cells.^38^ As significant differences in weekly body weight change and feed efficiency between the SCI groups were observed in the first‐week post‐treatment, we focused on levels of key pro‐inflammatory cytokines and GHs at a single time point 1‐week post‐treatment, when the changes in feed efficiency were observed. Although our current findings implicate the down‐regulation of systemic TNF‐α and IL‐6, and the upregulation of GHR and IGF‐1 in MSC‐sEV‐mediated recovery of growth after SCI, further evaluation of other inflammatory cytokines and anabolic hormones and other time points will be necessary to more fully understand potential growth regulatory mechanisms following SCI.

**FIGURE 8:**
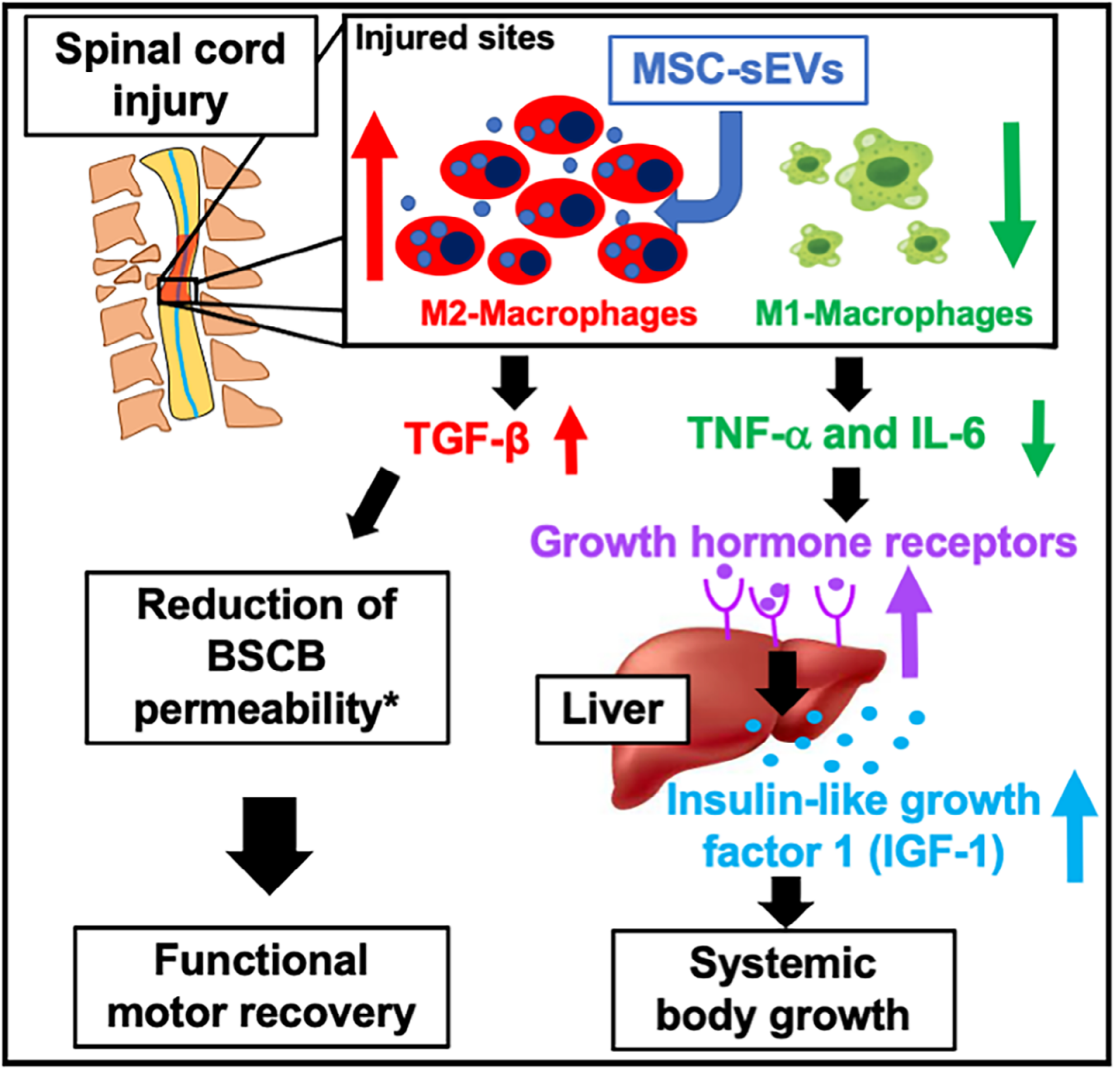
Proposed mechanisms underlying the therapeutic effects of small extracellular vesicles derived from mesenchymal stem/stromal cells (MSC‐sEVs) on restoring the body growth failure in spinal cord injury. IV‐delivered MSC‐sEVs are taken up by M2 macrophages at the lesion site and either prevent M1‐polarization around injured sites or increase M2 macrophage survival, resulting in decrease in the M1/M2 ratio at the lesion site. The decreased proportion of M1 macrophages leads to the down‐regulation of tumour necrosis factor‐alpha (TNF‐α) and interleukin (IL)‐6 production at the lesion site and a decrease in systemic levels of both TNF‐α and IL‐6 relative to spinal cord injury (SCI) alone. The decreased systemic levels of TNF‐α and IL‐6 result in a reduction in injury‐induced down‐regulation of growth hormone receptor (GHR) expression and greater production of insulin‐like growth hormone‐1 (IGF‐1) in the liver relative to the SCI alone. This increased IGF production results in greater systemic levels of IGF‐1 and restores the growth trajectory impaired by SCI. MSC‐sEVs uptake by macrophages also results in the upregulation of transforming growth factor‐beta (TGF‐β) at the lesion site, stabilization of the blood–spinal cord barrier and improvement of locomotor function.^10^ Hence, the uptake of MSC‐sEVs by macrophages at the lesion site results in upregulation of anti‐inflammatory cytokines and down‐regulation of pro‐inflammatory cytokines which act locally to facilitate tissue repair and functional recovery and systemically to recover growth hormone signalling and restoration of growth trajectory.

Previously, we proposed a mechanism of action for MSC‐sEV‐mediated improvement in function recovery after SCI whereby the uptake of MSC‐sEVs by M2 macrophages initiates a cascade of molecular responses that promotes the repair of the microvasculature and restoration of blood–spinal cord barrier integrity and produces an environment more conducive to neuronal repair and improved function.^10^ Here we propose that an M2 macrophage uptake of MSC‐sEVs may additionally mediate the therapeutic effects of MSC‐sEVs on the restoration of growth trajectory after SCI via reduction in systemic inflammation and restoration of GH/IGF signalling. Figure 8 We hypothesize that IV‐delivered MSC‐sEVs are taken up by M2 macrophages and stabilize the M2 anti‐inflammatory phenotype. The decreased proportion of M1 macrophages results in lower levels of TNF‐α and IL‐6 production at the lesion site and thereby decreases the systemic level of both TNF‐α and IL‐6. The lower levels of the serum of TNF‐α and IL‐6 following MSC‐sEV treatment result in less down‐regulation of GHR expression in liver, and therefore, higher production of IGF‐1. Elevated IGF‐1 allows a return towards a normal growth trajectory. Thus, a reduction in local inflammation at the lesion site promotes both neurological recovery and the restoration of a growth trajectory towards normal in SCI rodents treated with MSC‐sEVs.

The data presented here suggest a single mechanism may be responsible for both locomotor and growth recovery after SCI; however, as individual exosomes are too small to detect with conventional light microscopy, we cannot rule out the possibility that other cells in the spinal cord may have taken up exosomes below our threshold of detection. Furthermore, we have not yet examined the potential uptake of exosomes in the hypothalamus/pituitary gland or other hormone‐producing systems. Moreover, autofluorescence in the spleen made the potential uptake of exosomes in this tissue inconclusive. Hence, we cannot rule out the possibility that the body growth restoring effects of exosomes in SCI animals are not mediated by uptake by other hormone or cytokine‐producing cells. However, we have shown that exosome treatment is capable of promoting both improved locomotor recovery and restoration of growth trajectories and that the effects of exosomes on cytokine production by macrophages could explain both effects.

Early‐phase clinical studies have established the safety of single‐dose IV MSCs for both SCI^39^, ^40^ and stroke.^41^ However, IV cell therapies pose potential challenges in the clinical setting, including risks of pulmonary embolism and immuno‐rejection of nonautologous cells.^42^ These risks may limit the number of cells and treatment times. MSC‐sEV treatment represents a potentially attractive alternative to IV MSC therapy. Because of their much smaller size, sEV infusion presents a much lower risk of embolism. MSC‐sEVs have been demonstrated to have low immunogenicity in multiple clinical trials.^43^ They are also much more stable and storable than cells and offer the potential of producing a much more consistent treatment product which can be further modified to enhance therapeutic effects.^28^

Our findings comparing the therapeutic efficacy of human and rat MSC‐sEVs in a rodent SCI model show that rodents respond similarly to MSC‐sEVs derived from rat allogenic and human xenogeneic sources without immunosuppression. This represents an important step towards developing a cell‐related product for therapeutic applications. The responses of SCI rats to IV treatment of either human or rat MSC‐sEV treatment were not distinguishable, suggesting the validity of the SCI model to study treatment parameters including dosing levels and timing and mechanisms of IV MSC‐sEVs treatment.

## 5 CONCLUSIONS

The results of this study suggest a mechanism whereby intravenous MSC‐sEV treatment decreases systemic pro‐inflammatory cytokines resulting in increased GHRs and IGF‐1 in the liver that may contribute to the restoration of body growth which is interrupted after injury in young adult rats with SCI. Human‐derived MSC‐sEVs were as effective as rat‐derived MSC‐sEVs in promoting both functional recovery and normalization of body growth. This suggests that the interruption in growth after SCI in young rats can be mitigated by hMSC‐sEV treatment. These findings have important therapeutic implications for SCI patients and young SCI patients in particular where growth interruption after SCI may be more problematic.

## CONFLICT OF INTEREST STATEMENT

The authors declare that they have no competing interests.

## Supporting information

Supporting Information

Supporting Information

Supporting Information

Supporting Information

Supporting Information

Supporting Information
