# Supplementary material for: Human mesenchymal stem‐derived extracellular vesicles improve body growth and motor function following severe spinal cord injury in rat
Source: Clin Transl Med. 2023 Jun 15;13(6):e1284. doi: 10.1002/ctm2.1284 (PMC10272923; doi:10.1002/ctm2.1284)
Supplement: Supplementary file 1 — Supporting Information [file CTM2-13-e1284-s004.docx]

| **Supplementary Table 1. Information regarding the surgeries** | | | | | | | | |  |  |  |  |  |
| --- | --- | --- | --- | --- | --- | --- | --- | --- | --- | --- | --- | --- | --- |
| PBS | | | |  | hMSC-sEVs | | | |  | rMSC-sEVs | | | |
| Animal No | Body weight  (g) | Impact power (kilodynes) | Duration time (ms) |  | Animal No | Body weight  (g) | Impact power (kilodynes) | Duration time (ms) |  | Animal No | Body weight  (g) | Impact power (kilodynes) | Duration time (ms) |
| 1 | 198 | 229 | 15.06 |  | 1 | 198 | 235 | 15.09 |  | 1 | 200 | 240 | 16.32 |
| 2 | 200 | 252 | 16.18 |  | 2 | 198 | 244 | 15.08 |  | 2 | 202 | 231 | 16.01 |
| 3 | 212 | 230 | 16.34 |  | 3 | 200 | 290 | 15.39 |  | 3 | 198 | 240 | 16.00 |
| 4 | 214 | 229 | 15.71 |  | 4 | 217 | 238 | 16.03 |  | 4 | 193 | 229 | 16.02 |
| 5 | 212 | 250 | 15.69 |  | 5 | 205 | 227 | 16.31 |  | 5 | 220 | 231 | 16.32 |
| 6 | 208 | 230 | 16.00 |  | 6 | 192 | 226 | 15.38 |  | 6 | 208 | 248 | 15.69 |
| 7 | 200 | 227 | 16.03 |  | 7 | 219 | 275 | 16.00 |  | 7 | 200 | 227 | 15.37 |
| 8 | 214 | 234 | 15.71 |  | 8 | 206 | 229 | 15.07 |  | 8 | 200 | 274 | 16.02 |
| 9 | 201 | 242 | 15.06 |  | 9 | 210 | 239 | 15.39 |  | 9 | 198 | 262 | 16.01 |
| 10 | 223 | 228 | 16.00 |  | 10 | 200 | 260 | 15.06 |  | 10 | 191 | 289 | 15.08 |
| 11 | 205 | 242 | 16.02 |  | 11 | 220 | 236 | 16.02 |  | 11 | 203 | 230 | 16.63 |
|  |  |  |  |  | 12 | 221 | 229 | 16.32 |  | 12 | 215 | 230 | 16.01 |
|  |  |  |  |  |  |  |  |  |  |  |  |  |  |
| Average | 208 ± 2 | 236 ± 3 | 15.80 ± 0.12 |  |  | 207 ± 3 | 244 ± 6 | 15.60 ± 0.15 |  |  | 206 ± 2 | 244 ± 6 | 15.96 ± 0.12 |
|  |  |  |  |  |  |  | P value | | | |  |  |  |
|  |  |  |  |  |  |  | Body weight | Impact power | Duration time | |  |  |  |
|  |  |  |  | PBS vs. hMSC-sEVs | | | 0.979 | 0.508 | 0.524 | |  |  |  |
|  |  |  |  | PBS vs. rMSC-sEVs | | | 0.347 | 0.488 | 0.684 | |  |  |  |
|  |  |  |  | hMSC-sEVs vs. rMSC-sEVs | | | 0.437 | 0.999 | 0.135 | |  |  |  |
