# Supplementary material for: Human mesenchymal stem‐derived extracellular vesicles improve body growth and motor function following severe spinal cord injury in rat
Source: Clin Transl Med. 2023 Jun 15;13(6):e1284. doi: 10.1002/ctm2.1284 (PMC10272923; doi:10.1002/ctm2.1284)
Supplement: Supplementary file 2 — Supporting Information [file CTM2-13-e1284-s005.docx]

**Supplementary Table 2. Antibodies used in specific applications.**

| Primary Antibody (Manufacture, catalog #, dilution used) | Second Antibody (Manufacture, catalog #, dilution used) | Application |
| --- | --- | --- |
|  |  |  |
| Exosome specific protein makers |  |  |
| rabbit monoclonal anti-CD 9 antibody  (Abcam, ab92726, 1:1000) | Goat Anti-Rabbit IgG (H + L)-HRP Conjugate (BIO-RAD, 1706515, 1:25000) | Western blot analysis in Fig.1(C) |
| rabbit polyclonal anti-CD 63 antibody  (LSBio, LS-C408817, 1:1000) | Goat Anti-Rabbit IgG (H + L)-HRP Conjugate (BIO-RAD, 1706515, 1:25000) | Western blot analysis in Fig.1(C) |
| rabbit polyclonal anti-Alix antibody  (Proteintech,12422-1-AP, 1:1000) | Goat Anti-Rabbit IgG (H + L)-HRP Conjugate (BIO-RAD, 1706515, 1:25000) | Western blot analysis in Figure 1(C) |
|  |  |  |
| Macrophage makers |  |  |
| rabbit polyclonal anti-iNOS antibody  (Abcam, ab3523, 1:200) | Alexa Fluor 488-conjugated donkey anti-rabbit (Thermofisher, A21206, 1:1000) | Immunofluorescent M1 macrophages marker in Fig. 4(A), (B), (E), (F), 5(A), (B), 6(A^1^)-(C^1^), 6(A^3^)-(C^3^) |
| goat polyclonal anti-CD 206 antibody  (R&D Systems, AF2535, 1:200) | Alexa Fluor 594-conjugated donkey anti-goat (Thermofisher, A11058, 1:1000) | Immunofluorescent M2 macrophages marker in Fig. 4(A), (B), (E), (F), 5(A), (C), 6(A^2^-(C^2^), 6(A^3^)-(C^3^), Suppl. Fig. 1(A)-(C), (A^1^)-(C^1^) |
| mouse monoclonal anti-CD86 antibody  ( SANTA CRUZ, sc-376012, 1:50) | Alexa Fluor 488-conjugated goat anti-mouse (Thermofisher, A28175, 1:2000) | Immunofluorescent M1 macrophages marker in Suppl. Fig. 1(A),(B), (A^1^),(B^1^) |
| rabbit polyclonal anti-iNOS antibody  (Abcam, ab3523, 1:1000) | Goat Anti-Rabbit IgG (H + L)-HRP Conjugate (BIO-RAD, 1706515, 1:25000) | Western blot analysis in Fig. 6(D), (E), (G) |
| rabbit polyclonal anti-CD 206 antibody  (Abcam, ab64693, 1:500) | Goat Anti-Rabbit IgG (H + L)-HRP Conjugate (BIO-RAD, 1706515, 1:25000) | Western blot analysis in Fig. 6(D), (F), (G) |
|  |  |  |
| Others |  |  |
| mouse monoclonal anti-GHR antibody  ( SANTA CRUZ, sc-137185s, 1:100) | Anti-mouse IgG, HRP-linked Antibody (Cell Signaling, 7076, 1:5000) | Western blot analysis in Fig.7(E), (F) |
| rabbit monoclonal anti-GAPDH antibody  (Cell Signaling, 2118s, 1:20000) | Goat Anti-Rabbit IgG (H + L)-HRP Conjugate (BIO-RAD, 1706515, 1:25000) | Used as the inner control in Western blot analysis |
|  |  |  |
|  |  |  |
|  |  |  |
