# Supplementary material for: Human mesenchymal stem‐derived extracellular vesicles improve body growth and motor function following severe spinal cord injury in rat
Source: Clin Transl Med. 2023 Jun 15;13(6):e1284. doi: 10.1002/ctm2.1284 (PMC10272923; doi:10.1002/ctm2.1284)
Supplement: Supplementary file 3 — Supporting Information [file CTM2-13-e1284-s001.docx]

**Supplementary Table 3. PCR primer information used in this study.**

| Primer | TaqMan gene expression assay number | GenBank accession number | Figure |
| --- | --- | --- | --- |
| Tumor necrosis factor-alpha | Rn01525859_g1 | NM_012675.3 | Fig. 6(H) |
| IL-6 | Rn01410330_m1 | NM_012589.2 | Fig. 6(I) |
| Chemokine (C-C motif) ligand 2 (CLL2) | Rn00580555_m1 | NM_031530.1 | Fig. 6(J) |
| Transforming growth factor (TGF)-β1 | Rn00572010_m1 | NM_021578.2 | Fig. 6(K) |
| Mannose receptor, C type 1 (CD206) | Rn01487342_m1 | NM_001106123.2 | Fig. 6(L) |
| Arginase 1 | Rn00691090_m1 | NM_017134.3 | Fig. 6(M) |
| Growth hormone receptor | Rn00567298_m1 | NM_017094.1 | Fig. 7(D) |
| Insulin-like growth factor 1 | Rn00710306_m1 | NM_001082477.2 | Fig. 7(G) |
| Glyceraldehyde-3-phosphate dehydrogenase (GAPDH) | Rn01775763_g1 | NM_017008.4 | Inner control |
