# Supplementary material for: Human mesenchymal stem‐derived extracellular vesicles improve body growth and motor function following severe spinal cord injury in rat
Source: Clin Transl Med. 2023 Jun 15;13(6):e1284. doi: 10.1002/ctm2.1284 (PMC10272923; doi:10.1002/ctm2.1284)
Supplement: Supplementary file 4 — Supporting Information [file CTM2-13-e1284-s006.docx]

| **Supplementary Table 4. Data sets of body growth-related cytokines and hormones** | | | | | | |  |  |
| --- | --- | --- | --- | --- | --- | --- | --- | --- |
| No | Group | TNF-α (serum)  (pg/mL) | IL-6 (serum) (pg/mL) | GH (serum) (ng/mL) | GHR (gene) (Fold Change) | GHR (protein) (Normalized ratio) | IGF-1 (gene) (Fold Change) | IGF-1 (serum) (ng/mL) |
| 1 | Control | 2.075 | 116.669 | 91.902 | 1 | 1 | 1 | 326.8711 |
| 2 | Control | 9.228 | 68.19 | 87.204 | 1 | 1 | 1 | 392.2168 |
| 3 | Control | 0.01 | 139.68 | 67.476 | 1 | 1 | 1 | 396.7133 |
| 4 | PBS | 66.974 | 354.338 | 18.132 | 0.67 | 0.94387377 | 0.48 | 114.624 |
| 5 | PBS | 131.018 | 424.173 | 1.626 | - | 0.59700915 | - | - |
| 6 | PBS | 77.191 | 380.177 | 9.474 | 0.51733246 | 0.74385839 | 0.42631745 | 95.3278 |
| 7 | PBS | 256.595 | 430.347 | 1.872 | 0.22067575 | 0.43142235 | 0.21425334 | 70.6263 |
| 8 | PBS | 104.587 | 417.41 | 4.542 | 0.51763246 | 0.70477902 | 0.42044821 | 84.1427 |
| 9 | PBS | 90.075 | 408.857 | 2.964 | 0.49 | 0.68315734 | 0.33 | 81.5171 |
| 10 | PBS | 372.997 | 577.261 | 3.168 | 0.2102241 | 0.42 | 0.13 | 65.4637 |
| 11 | PBS | 350.079 | 458.034 | 9.198 | 0.49654625 | 0.45758913 | 0.35601255 | 72.7579 |
| 12 | PBS | - | - | 14.184 |  | 0.42141509 | - | - |
| 13 | PBS | - | - | 18.69 |  | 0.81504173 | - | - |
| 14 | PBS | - | - | 3.642 |  | 0.53949172 | - | - |
| 15 | PBS | - | - | 8.376 |  | - | - | - |
| 16 | hMSC-sEVs | 44.429 | 324.081 | 3.774 | 0.69737183 | 0.92678684 | 0.47963206 | 216.3162 |
| 17 | hMSC-sEVs | 77.191 | 331.822 | 5.724 | 0.60290391 | 0.79997615 | 0.49311635 | 123.0991 |
| 18 | hMSC-sEVs | 199.142 | 375.48 | 2.97 | 0.55864357 | 0.65689177 | 0.40895103 | 95.3278 |
| 19 | hMSC-sEVs | 34.436 | 306.788 | 5.028 | 0.61132014 | 0.83322388 | 0.54336743 | 218.8955 |
| 20 | hMSC-sEVs | 16.313 | 222.149 | 5.556 | 1.0942937 | 1.22397595 | 0.94605765 | 247.2575 |
| 21 | hMSC-sEVs | 92.462 | 350.13 | 26.82 | 0.59460356 | 0.74448779 | 0.43527528 | 116.809 |
| 22 | hMSC-sEVs | 21.446 | 243.585 | 8.31 | 1.07922824 | 1.19702151 | 0.6551967 | 240.6589 |
| 23 | hMSC-sEVs | 102.137 | - | 8.826 | - | 0.45879746 | - | - |
| 24 | hMSC-sEVs | - | - | - | - | 1.02857641 | - | - |
| 25 | rMSC-sEVs | 82.995 | 344.068 | 2.652 | 0.69255473 | 0.87954487 | 0.53588673 | 129.9084 |
| 26 | rMSC-sEVs | 62.533 | 317.835 | 13.302 | 0.73713461 | 0.98904472 | 0.52485834 | 168.5076 |
| 27 | rMSC-sEVs | 32.504 | 303.603 | 2.82 | 1.14869835 | 1.10470227 | 0.7631296 | 190.0632 |
| 28 | rMSC-sEVs | 91.267 | 414.568 | 29.628 | 0.56252924 | 0.77767236 | 0.40332088 | 105.2853 |
| 29 | rMSC-sEVs | 69.217 | 322.524 | 23.976 | 0.57038186 | 0.81956169 | 0.53589545 | 164.6584 |
| 30 | rMSC-sEVs | 90.075 | 351.639 | 13.02 | 0.57038186 | 0.816765 | 0.52123288 | 122.6483 |
| 31 | rMSC-sEVs | 58.158 | 243.585 | 31.074 | 1.08673486 | 1.01613696 | 0.62850669 | 175.1926 |
| 32 | rMSC-sEVs | 109.524 | - | 6.198 | - | 0.64525716 | - | - |
| 33 | rMSC-sEVs | - | - | - | - | 0.816765 | - | - |
