# Supplementary figures and images for: Human mesenchymal stem‐derived extracellular vesicles improve body growth and motor function following severe spinal cord injury in rat
Source: Clin Transl Med. 2023 Jun 15;13(6):e1284. doi: 10.1002/ctm2.1284 (PMC10272923; doi:10.1002/ctm2.1284)

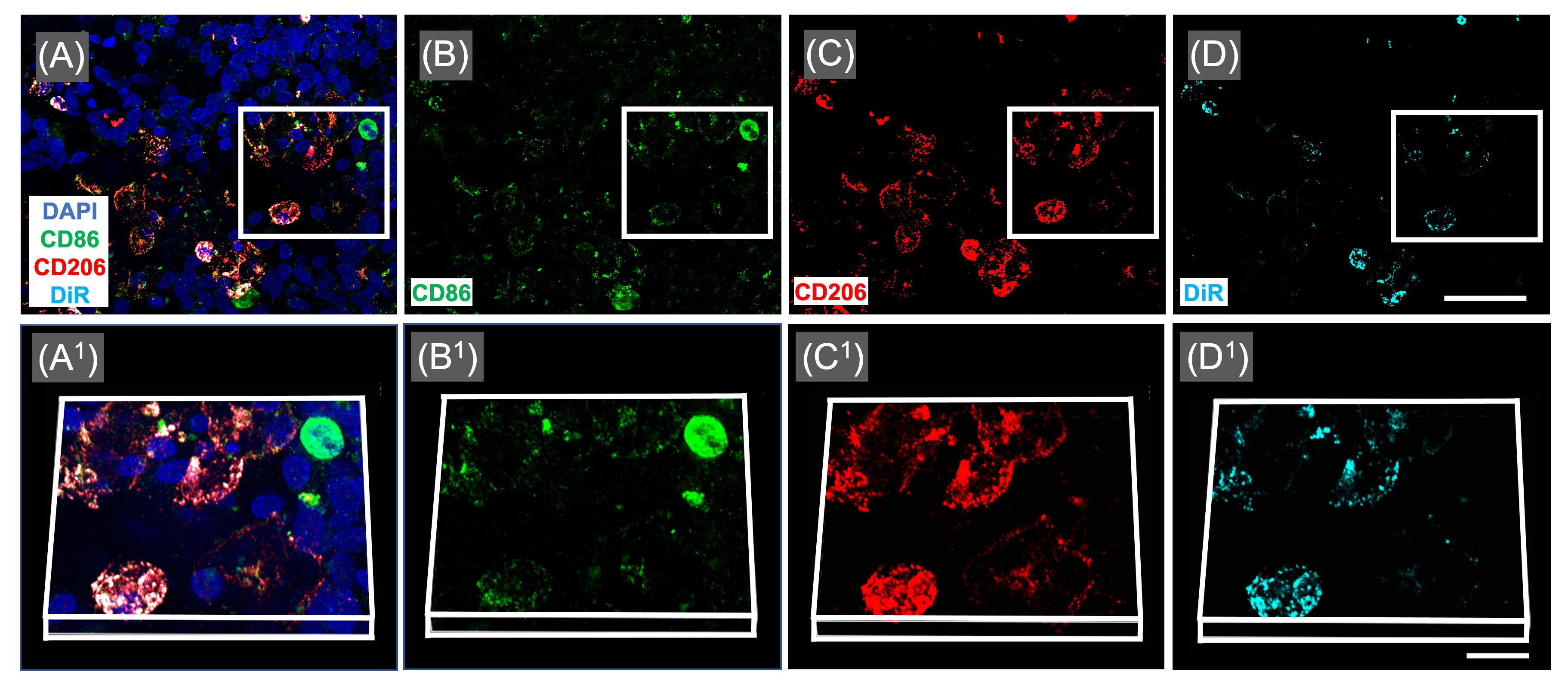

Supplement: Supplementary file 5 — Supporting Information [file CTM2-13-e1284-s002.tif]
