# Supplementary material for: Human mesenchymal stem‐derived extracellular vesicles improve body growth and motor function following severe spinal cord injury in rat
Source: Clin Transl Med. 2023 Jun 15;13(6):e1284. doi: 10.1002/ctm2.1284 (PMC10272923; doi:10.1002/ctm2.1284)
Supplement: Supplementary file 6 — Supporting Information [file CTM2-13-e1284-s003.docx]

Figure legends for Supplemental Figure 1

(A)-(D), Confocal micrographs of a representative region of a frozen sectioned contused spinal cord harvested 48 hours after IV infusion of DiR-labeled hMSC-sEVs, immunostained with antibodies directed against M1 macrophage maker CD86 (green), M2 macrophage marker CD206 (red) and DAPI (blue) with DiR visualized as cyan. Images show the same area showing fluorescence channels for (A), CD2D86, CD206, DAPI, & DiR, (B), CD86, (C), CD206, and (D), DiR. Images in (A^1^)-(D^1^) show enlarged images of the boxed area above rotated and illustrated in 3D. Scale bars in (A)-(D) and (A^1^)-(D^1^) indicate 20 μm and 10 μm, respectively. hMSC-sEVs: small extracellular vesicles derived from human mesenchymal stem/stromal cell, DiR: DilC18(7);1,1′-dioctadecyl-3,3,3′,3′-tetramethylindotricbocyanine iodide, DAPI: 4′,6-diamidino-2-phenylindole.
